# A Narrative-Gamified Mental Health App (Kuamsha) for Adolescents in Uganda: Mixed Methods Feasibility and Acceptability Study

**DOI:** 10.2196/59381

**Published:** 2024-12-19

**Authors:** Julia R Pozuelo, Christine Nabulumba, Doreen Sikoti, Meghan Davis, Joy Louise Gumikiriza-Onoria, Eugene Kinyanda, Bianca Moffett, Alastair van Heerden, Heather A O'Mahen, Michelle Craske, DoBAt & Ebikolwa Consortium, Munshi Sulaiman, Alan Stein

**Affiliations:** 1 Department of Global Health and Social Medicine Harvard Medical School Harvard University Boston, MA United States; 2 Department of Psychiatry University of Oxford Oxford United Kingdom; 3 MRC/Wits Rural Public Health and Health Transitions Research Unit (Agincourt) School of Public Health, Faculty of Health Sciences University of the Witwatersrand Johannesburg South Africa; 4 BRAC Kampala Uganda; 5 Mind Ease London United Kingdom; 6 College of Health Sciences Makerere University Kampala Uganda; 7 Mental Health Project Medical Research Council/Uganda Virus Research Institute (MRC/UVRI) Kampala Uganda; 8 Centre for Community Based Research Human Sciences Research Council Pietermaritzburg South Africa; 9 SAMRC/Wits Developmental Pathways for Health Research Unit, Department of Paediatrics, School of Clinical Medicine, Faculty of Health Sciences University of the Witwatersrand Johannesburg South Africa; 10 Mood Disorders Centre Department of Psychology University of Exeter Exeter United Kingdom; 11 Department of Psychology University of California, Los Angeles Los Angeles, CA United States; 12 Department of Psychiatry and Biobehavioral Sciences University of California, Los Angeles Los Angeles, CA United States; 13 See Acknowledgments Oxford United Kingdom; 14 BRAC University Kampala Uganda; 15 Africa Health Research Institute KwaZulu Natal South Africa; 16 Blavatnik School of Government University of Oxford Oxford United Kingdom

**Keywords:** adolescents, mental health, Uganda, gamified app, digital interventions, mobile phone, user-centered design, low- and middle-income countries

## Abstract

**Background:**

Many adolescents in Uganda are affected by common mental disorders, but only a few affordable treatment options are available. Digital mental health interventions offer promising opportunities to reduce these large treatment gaps, but interventions specifically tailored for Ugandan adolescents are limited.

**Objective:**

This study aimed to determine the feasibility and acceptability of the Kuamsha program, an intervention delivered through a gamified app with low-intensity telephonic guidance, as a way to promote mental health among adolescents from the general population in Uganda.

**Methods:**

A 3-month pre-post single-arm trial was conducted with adolescents aged between 15 and 19 years living in Wakiso District, Central Uganda. The intervention was coproduced with adolescents from the study site to ensure that it was culturally acceptable. The feasibility and acceptability of the intervention were evaluated using an explanatory sequential mixed methods approach. Feasibility was assessed by collecting data on trial retention rates and treatment adherence rates. Acceptability was assessed through a questionnaire and in-depth interviews with participants following the conclusion of the intervention period. As a secondary objective, we explored the changes in participants’ mental health before and after the intervention.

**Results:**

A total of 31 adolescents were recruited for the study. Results from the study showed high levels of feasibility and acceptability. Trial retention rates exceeded 90%, and treatment adherence was ≥80%. These results, evaluated against our predefined trial progression criteria, indicate a successful feasibility study, with all criteria exceeding the thresholds necessary to progress to a larger trial. App engagement metrics, such as time spent on the app and modules completed, exceeded existing literature benchmarks, and many adolescents continued to use the app after the intervention. In-depth interviews and questionnaire responses revealed high acceptability levels. Depressive symptoms trended toward reduction (mean difference: 1.41, 95% CI –0.60 to 3.42, Cohen *d*=0.30), although this was not statistically significant (*P*=.16). Supporting this trend, we also observed a reduction in the proportion of participants with moderate depressive symptoms from 32% (10/31) to 17% (5/29) after the intervention, but this change was also not significant (*P*=.10).

**Conclusions:**

This study presents evidence to support the Kuamsha program as a feasible and acceptable digital mental health program for adolescents in Uganda. A fully powered randomized controlled trial is needed to assess its effectiveness in improving adolescents’ mental health.

## Introduction

### Background

The prevalence of common mental disorders among adolescents in Uganda is alarmingly high, with estimates reaching up to 25% [1-3]. When left unaddressed, common mental disorders such as depression and anxiety can profoundly impact the developmental trajectories of adolescents, with potentially long-lasting consequences that extend into adulthood [4]. Furthermore, the pervasive conditions of poverty, coupled with daily stressors such as exposure to violence and malnutrition, make adolescents in low-income communities more susceptible to developing mental health disorders [5,6].

Common mental disorders are underdiagnosed and undertreated globally, particularly in low- and middle-income countries, where there is minimal public expenditure on mental health (US $0.02 per person/y) and an acute shortage of skilled mental health professionals, especially in rural areas [7-10]. Further obstacles to accessing treatment include low levels of mental health literacy and high levels of associated stigma [11-13]. Given the high prevalence and enduring consequences of depression and anxiety, it is critical to develop scalable and cost-effective strategies to improve adolescent mental health in low-income countries like Uganda.

As smartphone ownership and internet access continue to rise globally, especially among adolescents, digital solutions offer a promising way to help overcome various obstacles to expanding mental health services [14]. Digital mental health interventions offer a range of benefits, enabling broad reach without substantial per-user expense [15]. They can be accessed remotely and provide flexibility and convenience to users, who can discreetly access them where and when they choose, enhancing accessibility and reducing stigma-related barriers. Furthermore, by providing an alternative to traditional in-person treatments, digital mental health interventions can help alleviate the strain on overloaded health care systems.

Despite the many potential advantages of digital mental health interventions, studies on their effectiveness in adolescents have yielded mixed results [16-18]. This variability in outcomes can be partly attributed to the low levels of user engagement and high dropout rates characterizing these interventions. For instance, 96% of users abandon mental health apps within just 15 days of downloading [19]. While a few commercial smartphone apps attract more users, many lack rigorous evaluation and have limited fidelity to evidence-based treatments [20,21]. Furthermore, most available evidence comes from high-income countries, with few digital interventions deliberately developed and evaluated in low-income settings such as Uganda [14]. The effectiveness of these interventions in these settings is likely compromised, as research consistently demonstrates that interventions tailored to fit the cultural, linguistic, and environmental contexts of their target audience are more effective [22]. This highlights the critical need for integrating localized insights and user feedback early in the development process, ensuring interventions are aligned with the needs and realities of their intended users.

### Study Rationale and Objectives

This study, conducted in Uganda and in collaboration with its counterpart study in rural South Africa, aimed to address these limitations by developing a digital intervention that not only fostered active user engagement but also adhered to evidence-based principles while maintaining relevance and relatability to the target population. With this vision in mind, we developed the Kuamsha program, a narrative-gamified mobile app delivering behavioral activation therapy, an evidence-based treatment for depression that has been adapted as a general emotional well-being intervention for adolescents from the general population [23,24]. The Kuamsha app was designed to be supported with weekly phone calls from peer mentors. The development process, previously described in another publication [25], involved an iterative co-design process with 160 adolescents and other stakeholders in Uganda and South Africa.

The primary objective of this study was to evaluate the feasibility and acceptability of the Kuamsha program among adolescents from the general population in Uganda. To assess this, we used an explanatory sequential mixed-methods design, whereby we initially gathered quantitative data on the intervention’s performance, followed by qualitative data to elucidate these findings. The secondary objective was to explore changes in participants’ mental health (depressive symptoms, anxiety, and emotional well-being) by comparing baseline assessments with those conducted at the end of the intervention.

## Methods

### Study Design

This study was a 3-month pre-post single-arm trial. Participants were recruited between November and December 2021, and in-depth interviews were conducted after the intervention between April and May 2022.

### Study Setting

The study was conducted in catchment areas mapped around Katabi town in Wakiso District, Central Uganda. Katabi town represents a periurban area, located approximately 33 km from Kampala and 11 km from Entebbe. The site combines periurban communities close to the Entebbe-Kampala Road with relatively isolated fishing villages, which stretch to the shores of Lake Victoria.

The study area is socioeconomically disadvantaged, with subsistence farming as the predominant occupation. Approximately 50% of adolescents in this area attend secondary school, while 15% of young individuals (aged between 18 and 30 y) are neither employed nor engaged in formal education. Literacy rates of individuals aged between 10 and 30 years are above 87%. The nearest specialist psychiatric resources are in Kampala, a 2-hour journey by public transportation [26]. Data from the National Population and Housing Census in Uganda suggest that >80% of adolescents in Wakiso District own a mobile phone, and this is increasing steadily [26].

### Study Participants

To be eligible to participate, respondents had to be aged between 15 and 19 years at the time of recruitment; be willing and able to give informed consent (for participants aged ≥18 years or emancipated adolescents) or assent (if aged <18 years); have a caregiver willing to provide consent (if aged <18 years); have an intention to reside in the area for 12 weeks following enrollment; and demonstrate fluency and literacy in English or Luganda, as confirmed by a score of 83% (5/6 correct answers) or higher on a reading comprehension assessment adapted from the Young Lives study [27].

### Study Procedures

We used a systematic random sampling methodology to identify eligible participants [28]. By starting at a random location for each cluster and selecting a direction, field-workers visited every fifth house and determined if there were any eligible adolescents. When identified, field-workers provided eligible participants and their caregivers (if adolescents were aged <18 years and not emancipated) with an information sheet and arranged another visit at least 24 hours later to review it and obtain informed consent. For participants aged <18 years, the field-worker took informed consent from the caregivers during the first screening visit, if available, or during the second visit. If a house contained >1 eligible adolescent, we applied the Kish method to select the individual randomly.

After obtaining informed consent or assent, fieldworkers interviewed the adolescents at their homes in a private space using a structured baseline questionnaire. During the same visit, adolescents were provided with a low-end Samsung smartphone that had the Kuamsha app preinstalled as well as all its modules (ensuring accessibility to the content irrespective of an internet connection).

There were 2 in-person assessments, including the baseline assessment (week 0) and the end of intervention (week 11). In addition to these assessments, participants received active symptom monitoring via SMS text messages sent to the smartphone at weeks 2.5 and 7.5.

After the intervention, a separate qualitative field-worker visited all adolescents on another occasion to complete an in-depth interview. All interviews were conducted in Luganda by experienced Luganda-speaking qualitative field-workers. Semistructured interview methods were developed to elicit data on participants’ experience with the Kuamsha program, including their perceptions of the app, the peer mentor component, and the overall usability and impact of the intervention on their daily lives (Multimedia Appendix 1). Qualitative field-workers were trained in qualitative interviewing by CN and DS. Interviews had an average duration of 20 (SD 4) minutes.

The survey instruments and app content were translated and back translated into Luganda by a team of professional translators and were checked by a Ugandan bilingual clinical psychologist for linguistic and cultural accuracy. While enumerators conducted interviews, data for sensitive questions were collected using computer-assisted self-interviewing audio software, where participants listened to prerecorded audio of the survey questions via headphones and selected their responses on a tablet screen.

### Intervention

**Figure 1:**
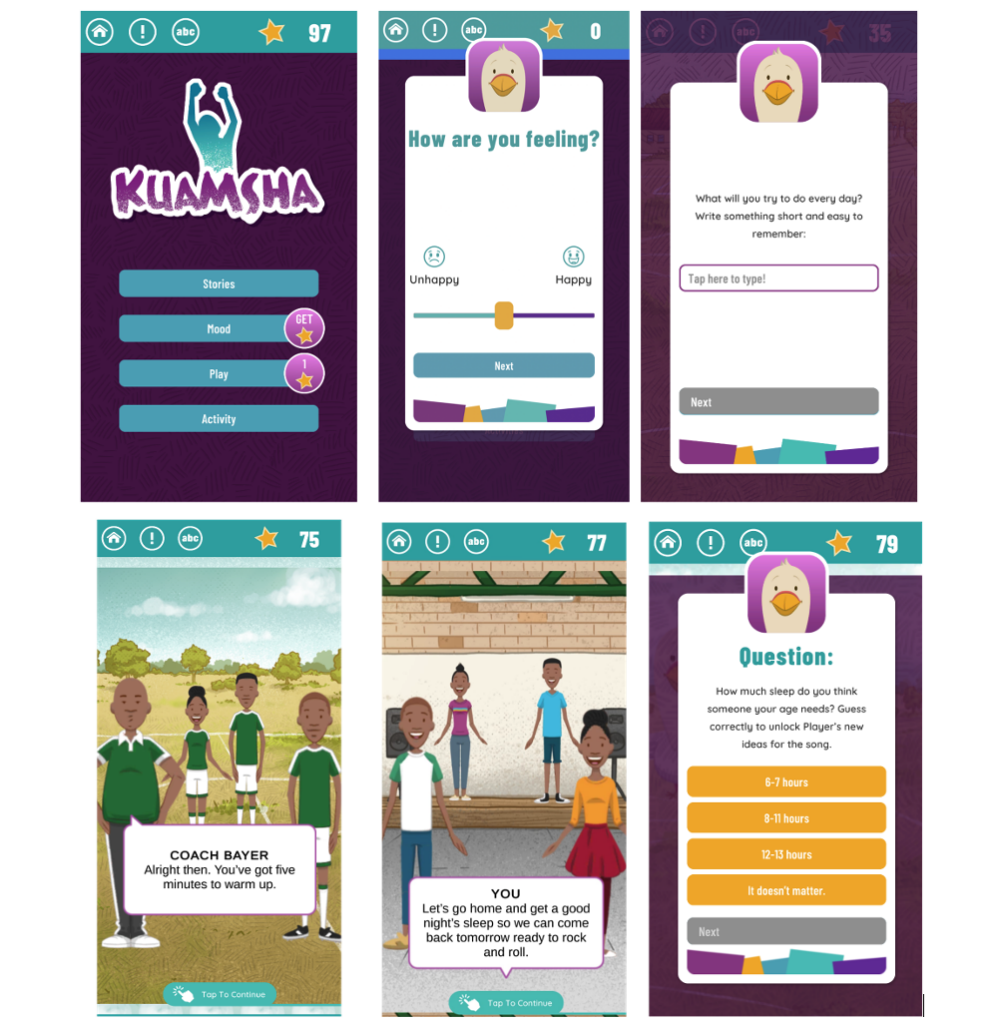
Kuamsha app wireframes.

Kuamsha is a gamified smartphone app that delivers behavioral activation therapy using choose-your-own-adventure narrative stories, which are a popular genre of games in the app store [25]. The core of the game consists of a choice between 2 stories, and each consists of 6 modules (labeled as “episodes”) that are played in sequential order. Adolescents were encouraged to complete 6 modules over the 11-week treatment phase. Each module takes approximately 15 to 20 minutes to complete and covers different behavioral activation skills, such as activity scheduling, avoiding the traps of negative thoughts, and getting enough sleep. After completing each module, users were asked to complete homework activities related to the skill learned in the module. Users were also encouraged to report the number of times they completed the homework they set for themselves and to monitor their mood as they were doing these activities. The app also incorporates regular mood check-ins and gaming elements, including in-app points, personalization, adjustable difficulty levels, and push notifications. Example wireframes of the app and its main components are depicted in Figure 1, with additional wireframes available in the Multimedia Appendix 2.

Each participant was paired with a trained peer mentor, whose role was limited to helping users understand the app’s content and overcome barriers to engagement. Adolescents received up to 7 calls in total, including 1 introductory phone call and 6 calls to cover module content. To ensure consistency and adherence to the study protocol, peer mentors conducted weekly calls based on a predefined fidelity checklist. The details of the training and supervision of the peer mentors, along with all measures and tests used, will be described and made available in a separate publication.

### Outcomes

#### Primary Outcome Measures: Feasibility and Acceptability

The feasibility and acceptability of the intervention were evaluated using a mixed methods approach.

#### Feasibility

Feasibility was assessed by collecting data on 2 aspects.

The first aspect was *retention in the study* at the end of the intervention period (11 weeks). This was directly linked to our trial progression criteria (Table 1), where retention rates of ≥90% were considered within the green zone, rates between <90% and ≥50% fell into the amber zone, and rates below 50% were categorized as red.

The second aspect was *treatment adherence rates*, where adherence is defined as a participant having opened ≥4 of the 6 app modules and as a participant having completed 3 of the 6 phone calls with the peer mentor (excluding the introductory call that did not cover any module content). Adherence was also part of our trial progression criteria, which delineated green zone for adherence levels of ≥70%, amber zone for levels between <70% and ≥50%, and red zone for levels below 50%. The treatment adherence rates were complemented with engagement metrics collected via the app and included the frequency of app log-ins, module completion rates, total time spent on the app, the number of weekly activities set to do, and the frequency of completed weekly activities.

**Table 1:** Trial progression criteria.

Criteria	Green zone (%)	Amber zone (%)	Red zone (%)
Study retention at 11 weeks	≥90	<90, ≥50	<50
Share of participants that open ≥4 of the 6 app modules	≥70	<70, ≥50	<50
Share of participants that have 3 of 6 phone calls with the peer mentor (excluding introductory call).	≥70	<70, ≥50	<50

#### Acceptability

Acceptability of the intervention and study procedures was assessed through 2 methods.

In the first method, an acceptability questionnaire was conducted at the postintervention assessment with all participants. The questionnaire consists of 3 measures: acceptability of intervention, intervention appropriateness, and feasibility of intervention [29]. Each measure comprised 4 items, with the total score ranging from 1 to 5, calculated by averaging the response scores to the response categories. An average score was calculated for each of the measures.

In the second method, in-depth interviews with all participants about their experience in the Kuamsha program were conducted.

#### Fidelity

The fidelity of delivery of the intervention was assessed through 2 methods. The first method was the adherence of the peer mentors, defined as the number of sessions that met at least 90% of the criteria for adherence according to the training protocol. In total, 2 independent raters listened and rated one-third of all peer mentors’ phone calls stratified by peer mentors and the treatment phase. Each call was scored using a specifically developed supervisor feedback form, which evaluated competence and adherence across 6 domains, including professionalism, ethical standards, cultural sensitivity, reflective practice, problem-solving, and participant engagement. The form also included a fidelity checklist to verify whether each call was executed timely, lasted between 15 and 20 minutes, and covered key topics. Initially, the raters independently evaluated 8 calls to calibrate their scoring. Once they reached a consensus on their scoring approach, they proceeded to independently assess the remaining calls, selected randomly, across several weeks.

The second method was the competence of the peer mentors, expressed as a percentage based on their competency assessment test. Conducted immediately after the training, this test included a written test and observation of skills through role-playing. Tests were scored by the peer mentor supervisor (JLGO) using a predetermined scoring system.

#### Secondary Outcome Measures: Changes in Mental Health

We used the following measures to evaluate changes in participants’ mental health:

##### Depressive Symptoms

Depressive symptoms were measured by the Patient Health Questionnaire Adolescent version (PHQ-A). The PHQ-A is a well-established measure to assess depressive symptoms over the preceding 2 weeks [30]. The PHQ-A total score ranges from 0 to 27. A score of 1 to 4 indicates minimal depression, 5 to 9 suggests minor depression, 10 to 14 corresponds to moderate depression, 15 to 19 indicates moderately severe depression, and a score of 20 to 27 reflects severe depression [31]. The PHQ-9 has been used in Uganda and has shown good psychometric properties among adolescents [32]. Participants completed this scale at baseline (week 0), at the end of the intervention (week 11), and as part of the symptom monitoring (weeks 2.5 and 7.5).

##### Emotional Well-Being

Emotional well-being was measured by the Warwick-Edinburgh Mental Wellbeing Scale (WEMWBS). The WEMWBS is a 14-item questionnaire to assess psychological functioning and emotional well-being [33]. The questionnaire has shown good validity and reliability across various cultural and geographical groups, including in Uganda and other sub-Saharan African contexts [34,35]. The WEMWBS total score for the full-scale ranges from 14 to 70, with high scores indicating a higher level of mental well-being. Participants completed this scale at baseline (week 0) and at the end of the intervention (week 11).

##### Anxiety Symptoms

Anxiety symptoms were measured by the Generalized Anxiety Disorder-7 (GAD-7). GAD-7 is a validated 7-item tool to assess symptoms of generalized anxiety disorder over the previous 2 weeks [36]. The GAD-7 total score ranges from 0 to 21, with higher scores representing increased anxiety (0-5 mild; 6-10 moderate; 11-15 moderately severe anxiety; and 15-21 severe anxiety). Participants completed this scale at baseline (week 0) and at the end of the intervention (week 11). GAD-7 has been used previously in Uganda [37-39].

#### Sociodemographics

##### Poverty

We used a household asset index designed for Uganda to estimate the likelihood of households falling below the poverty line [40]. The tool consists of 10 verifiable items on household assets and characteristics. Total scores range from 0 (most likely below a poverty line) to 100 (least likely below a poverty line).

##### Food Insecurity

We assessed this using the 6-Item Short Form of the Household Food Security Scale [41]. The tool measures food insecurity and hunger within the last 12 months. Respondents indicated how frequently they encountered situations such as running out of food or skipping meals. Food security status is determined based on raw scores: 0 to 1 indicates high or marginal food security, 2 to 4 denotes low food security, and 5 to 6 signifies very low food security.

##### Study Progression Criteria

We used predefined trial progression criteria to decide whether to proceed to a larger trial [42]. These criteria, outlined in Table 1, were structured into 3 distinctive categories, each associated with specific thresholds. The green zone recommends continuation to a larger pilot study, the amber zone indicates the need for cautious advancement with modifications, and the red zone suggests discussions with the study team about not progressing to a larger pilot study.

### Analysis

Quantitative data were analyzed using Stata (version 17.0; StataCorp) [43]. For each quantitative outcome, we computed appropriate summary statistics (eg, proportions, means, SDs, etc). Mean changes in mental health scores at baseline and 11-week assessment were estimated using paired, 2-tailed *t* tests. Statistical significance was set at *P*<.05. These *t* tests were interpreted cautiously as this study was not powered to detect statistically significant differences.

Qualitative in-depth interviews were audio recorded, transcribed verbatim, and translated from Luganda into English. The anonymized transcripts were imported into NVivo (version 12.0; Lumivero) software and analyzed using thematic analysis [44]. The data analysis team consisted of researchers with diverse perspectives, including those with extensive experience in mental health in Uganda and others new to working with this community, encompassing a range of backgrounds and experiences.

Data analysis was conducted in phases. In the first phase, 2 researchers (DS and MD) independently coded 5 interviews to develop a preliminary coding framework focused on identifying barriers and facilitators of the digital intervention. Inductive coding was also used to capture additional themes emerging from participants’ narratives. Codes were refined and organized into broader categories, such as grouping smartphone issues, app bugs, and mobile data challenges under the overarching category of *technical issues*. A codebook was developed with definitions and examples of codes and categories to guide subsequent analysis. This refined coding scheme was applied to all remaining interviews, with researchers meeting regularly to resolve discrepancies, reach consensus, and ensure consistency across the dataset. These meetings facilitated further refinement of the codebook by adding, merging, or clarifying codes as needed. Representative quotations that reflected key themes were selected for inclusion in the manuscript.

Reflexivity was integrated into the analysis through reflexive memoing, allowing researchers to reflect on their assumptions and biases. Documentation of coding decisions and analytical reflections strengthened the confirmability of findings. Regular coder meetings and the use of NVivo software contributed to a structured and transparent coding process, ensuring dependability. Credibility was strengthened through cross-coder verification and discussions with the broader research team, which included mental health professionals, peer mentors, and individuals familiar with the local context. This team provided feedback, reviewed findings, and suggested refinements, fostering reflexivity and enriching data interpretation.

Data saturation was reached after approximately 20 interviews, with no new themes emerging in subsequent interviews. This aligns with previously reported findings [45], indicating that our sample size was sufficient to capture the primary themes related to intervention acceptability and feasibility.

### Ethical Considerations

The study was reviewed and approved by institutional review boards in Uganda (Makerere University School of Public Health and the Uganda National Council for Science and Technology, with reference numbers HDREC750 and HS72 4ES, respectively) and the United Kingdom (Oxford Tropical Research Ethics Committee, OxTREC 72-19). Before recruitment, we obtained verbal approval from the local council chairperson and community elders. Written informed consent was obtained from either the parent or legal guardian of adolescents aged between 15 and 17 years or directly from adolescents aged ≥18 years or emancipated adolescents. In addition, written assent was obtained from participants aged <18 years. Participant data were anonymized by assigning unique participant ID numbers at recruitment. All participants were provided with a smartphone, which they could keep at the end of the study. Although the app was designed to function without internet connectivity, participants were also provided with smartphone data. This ensured that data from the app could be uploaded to the server for analysis. They did not receive any other compensation for participation in the study.

## Results

### Baseline Characteristics

Characteristics of study participants at baseline are shown in Table 2 for all 31 adolescents who were enrolled in the feasibility study. We observed a high prevalence of elevated depressive and anxiety symptoms among the recruited adolescents. For our sample, 32% (10/31) of participants exhibited moderate depressive symptoms (PHQ-A score ≥10), and 13% (4/31) had moderate to severe anxiety symptoms (GAD-7 score ≥10). This prevalence is similar to that found in meta-analyses, where the pooled prevalence of depression was 23.6% and anxiety disorders were 14.4% among children and adolescents in Uganda [46,47]. Further details of the distribution of these variables can be found in Multimedia Appendix 3.

**Table 2:** Characteristics of study participants at baseline (n=31).

Characteristics		Values
**Sociodemographic characteristics**		
	Age (y), mean (SD)	17.58 (1.43)
	Female, n (%)	15 (48)
	Married, n (%)	2 (6)
	Enrolled in school, n (%)	27 (87)
	Years of education, mean (SD)	9.65 (2.04)
	Worked in the past 7 days, n (%)	11 (58)^a^
	Household asset index, mean (SD)	58.57 (10.77)
	Food insecurity, n (%)	14 (61)^b^
**Mental health characteristics, mean (SD)**		
	Depression symptoms: PHQ-A^c^ score	6.52 (4.76)
	Emotional well-being: WEMWBS^d^ score	47.68 (12.21)
	Anxiety symptoms: GAD-7^e^ score	4.39 (4.69)

^a^Data on “Worked in the past 7 days” is available for 19 adolescents.

^b^Data for food insecurity is only available for 23 adolescents.

^c^PHQ-A: Patient Health Questionnaire Adolescent version.

^d^WEMWBS: Warwick-Edinburgh Mental Wellbeing Scale.

^e^GAD-7: Generalized Anxiety Disorder-7.

High levels of poverty were observed, as the household asset index scores between 55 and 59 corresponded to a 27.9% likelihood of living below the international poverty line of US $3.10/day (purchasing power parity; 2011 prices). Food insecurity was prevalent among participants.

### Primary Outcome Measures: Feasibility and Acceptability

#### Feasibility

##### Retention in the Study

**Figure 2:**
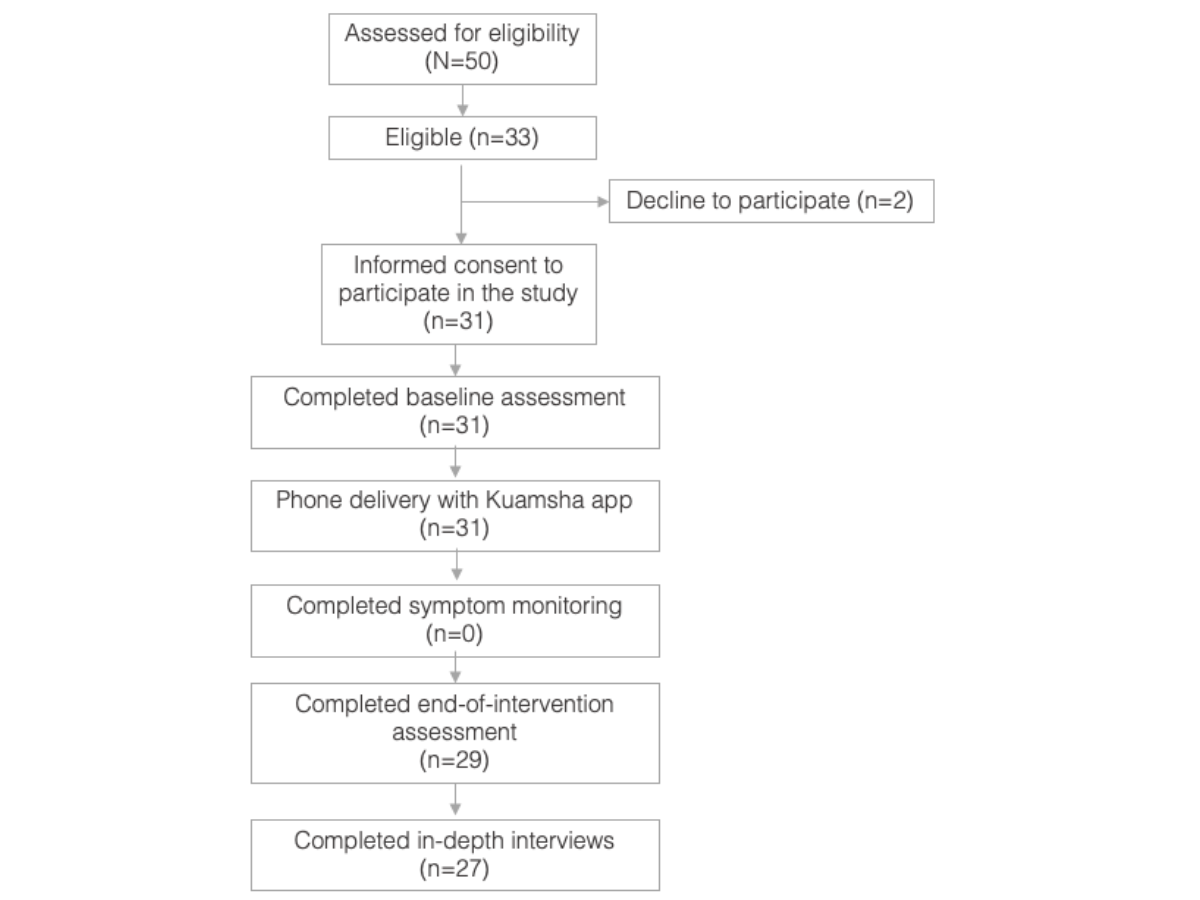
Study flow.

Figure 2 depicts that we had high retention rates during the study, with 94% (29/31) of enrolled participants retained until the end of the intervention assessment. In total, 6% (2/31) participants declined this final assessment due to lack of time, although both had engaged with the intervention (completing ≥6 modules and 2 peer mentor calls). In addition, 7% (2/29) of participants who completed the end of the intervention assessment were unavailable for interviews as they moved out of the study area to attend boarding school. Overall, this retention rate places the study well within the green zone according to our previously defined trial progression criteria, which set a benchmark for proceeding to a larger trial at a retention rate of ≥90%.

As depicted in Figure 2, the SMS text message–based symptom monitoring system encountered significant feasibility issues, primarily due to the need for stable internet connectivity for both sending out messages and receiving incoming data. Efforts to synchronize the distribution of airtime with the timing of these SMS text messages were unsuccessful. In addition, the onset of the COVID-19 pandemic further complicated this aspect of the study, ultimately leading to the decision to discontinue the symptom monitoring system.

##### Treatment Adherence Rates

The app metrics indicated high levels of engagement (Table 3). Notably, 97% (30/31) of participants opened ≥4 app modules, and 81% (25/31) completed ≥3 phone calls with the peer mentors. Both metrics place user engagement within the green zone, surpassing our trial progression criteria that mandated a ≥70% adherence rate for both app use and peer mentor calls.

**Table 3:** Treatment engagement (n=31).

			Values
**Treatment adherence, n (%)**			
	Opened ≥4 app modules	30 (97)	
	Had ≥3 phone calls with peer mentors	25 (81)	
**App engagement metrics, mean (SD)**			
	Log-ins	38.90 (32.33)	
	Modules opened	19.52 (17.96)	
	Modules completed	12.29 (12.93)	
	Total time spent on the app	6 h 09 min (5 h 52 min)	
	Completed weekly activities	91 (116.72)	
**Peer mentor engagement metrics (total=179 calls)**			
	Phone call duration (min), mean (SD)	16.03 (3.35)	
	Time days between phone calls, mean (SD)	10.77 (5.74)	
	Participants who completed all phone calls, n (%)	20 (65)	

However, there was considerable heterogeneity in how adolescents engaged with the Kuamsha app, as indicated by the SDs (Table 3) and the distribution of key app metrics (Multimedia Appendix 4). For example, some (6/31, 19%) participants logged into the app an average of >8 times per day, while 1 (3%) participant logged in twice over the entire treatment phase. Similarly, approximately one-third (12/31, 39%) of the participants completed all available modules, while approximately a quarter (7/31, 23%) of them did not meet the recommended dose of 6 modules. This variation also extended to the type of content explored. Most (21/31, 68%) participants explored both stories, while 16% (5/31) exclusively engaged with the song contest story, and 13% (4/31) focused on the football match story only. However, 1 (3%) participant did not open either story. These findings suggest that a “one size fits all” digital approach may not suit all participants, a point further explored in the Discussion section.

Despite this variability, the results showed that users, on average, surpassed the recommended treatment dose of 6 modules over the 11-week treatment phase, with an average completion of 12.29 (SD 12.93) modules. However, some (10/31, 32%) participants initially rushed through the modules, particularly when they were first given the phones. This prompted peer mentors to encourage a structured pace, advising them to complete 1 module per week to ensure they had sufficient time for the accompanying homework activities.

Participants spent approximately 6 hours on the Kuamsha app, on average. Almost half (14/31, 45%) of the participants continued to use the app even after the study had ended. Adolescents chose a range of goals to work on during the intervention period. The most frequently chosen goals included helping with household chores, reading books, going to bed early every night, studying, exercising, and trying to make new friends. In addition, some participants mentioned singing and playing football, which may have been inspired by the stories in the app. Some adolescents chose goals that were not clearly defined, quantifiable, or feasible to achieve within the intervention period (eg, “I want to live a happy life,” “Be successful,” and “Become a doctor”). Peer mentors received training to help refine these goals during the weekly calls.

The number of phone calls with the peer mentors also varied, with a median of 6 (IQR 2-6) calls and 2 (6%) of the 31 participants completing only 1 call. There was a positive correlation between the frequency of phone calls with peer mentors and app engagement. Those who engaged in more calls were more likely to log in to the app (standardized β coefficient=0.41, *P*<.05), suggesting that peer mentors played a key role in boosting app engagement.

#### Acceptability

##### Acceptability Questionnaire

Table 4 presents findings related to participants’ perceptions of the Kuamsha program at the end of the intervention in terms of acceptability (acceptability of intervention measure), appropriateness (intervention appropriateness measure), and feasibility (feasibility of intervention measure).

The consistently high scores across all categories reflect a strong positive reception from participants. However, this also makes it challenging to discern meaningful variations or discrepancies in the participants’ responses, a challenge that the in-depth interviews help overcome by providing a more granular understanding of the intervention’s acceptability.

**Table 4:** Acceptability questionnaire (n=29).

			Values (range 1-5), mean (SD)
**Kuamsha app**			
	Acceptability (AIM^a^)	4.33 (0.58)	
	Appropriateness (IAM^b^)	4.34 (0.56)	
	Feasibility (FIM^c^)	4.35 (0.49)	
**Peer mentor calls**			
	Acceptability (AIM)	4.32 (0.61)	
	Appropriateness (IAM)	4.28 (0.61)	
	Feasibility (FIM)	4.24 (0.63)	

^a^AIM: acceptability of intervention measure.

^b^IAM: intervention appropriateness measure.

^c^FIM: feasibility of intervention measure.

##### In-Depth Interviews

###### Overview

We conducted in-depth interviews with 27 (93%) of the 29 adolescents who completed the intervention (female adolescents: n=13, 48% and male adolescents: n=14, 52%). Overall, we identified 5 main themes around facilitators to the intervention and 4 main themes around barriers. A summary of key identified facilitators is presented in Table 5, and a summary of key barriers is shown in Table 6.

**Table 5:** Summary of key identified facilitators.

Theme and subtheme		Quotes
**User-friendly app interface**		
	The app was enjoyable and useful	“It teaches, encourages, motivates. I gained patience. I gained a lot.” [P14, female] “The app is going to help in keeping me busy from doing things that can make me get off track...like drinking alcohol and smoking cigarettes.” [P4, male]
	Easy to use	“Like its graphical interface, the way it was appearing was not complicated. It’s easy to use, and it has simple language.” [P1, male]
**Technical characteristics**		
	Convenience and offline functionality	“The app is so simple it doesn’t need airtime, and it didn’t have a specific time that you should play it. It’s available every time.” [P5, male]
**Cultural validity and gamification**		
	Narrative game format	“The way they were narrating the story it is understood in a way that it touches you and gives you a feeling that is different.” [P22, male] “It’s like you play and learn at the same time.” [P1, male]
	Relatable stories	“What you read, it was like going direct to your heart” [P29, male] “The graphics were like real, and the activities relate to our behavior in society...it was really relating to me.” [P6, male]
	Uplifting	“Playing those stories...made me happier.” [P30, male] “The app takes my stress away.” [P22, male; P10, male]
**Peer mentors’ encouragement**		
	Empathic approach	“The peer mentor was a very good person in that he encouraged me...When I had a problem, he could ask me about it, and we solve it together. With time, the peer mentor became my friend.” [P6, male]
	Emotional support	“Sometimes she called when I am not in a good mood, but I would not show her that I would talk calmly with her and sometimes by the time I finished talking to her whatever was disturbing me would have disappeared.” [P26, female] “Whenever he could notice my anger, he could call me by name and asked me to be calm and indeed I could calm down and feel happy on my heart. He was almost like my Dad...he was always there to follow up on me.” [P29, male]
	Peer mentors supported adolescents with the app	“The peer mentor gave me advice about how to use the app...I used to skip the episodes or did all the episodes at once, and she was like, you have to play one episode in a week.” [P16, female] “At first, I wasn’t using the app well, but when the peer mentor called me and asked me have you done this or that, I remembered that there are things that I had forgotten to do and I started to correct them.” [P5, male]
**Peer mentors flexibility**		
	Scheduling calls in advance and flexibility in rescheduling	“We usually agreed on the time and day to call...and she would call at the time. So, it was easy.” [P24, female] “Even when already talking to her and you are interrupted, she will give you time to sort the issue and get back to the call.” [P26, female]

**Table 6:** Summary of key identified barriers.

Theme		Quotes
**Comprehension and literacy skills**		
	Difficulty comprehending the app’s content	“I would have played better...if there was someone who would read for me the words because it is hard for me to read.” [P21, female] “The time it took for reading is hard because I am not interested in reading.” [P7, male] “Sometimes when you are not in a good mood...what you read on the app might not make much sense to you.” [P20, female]
**Technological glitches**		
	Looping bug	“Sometimes when I opened, I had to play the mood like 15 times...and it used to annoy me.” [P16, female]
**Logistical problems scheduling the peer mentor calls**		
	Finding a convenient time to talk	“It was difficult most of the times for the reason being there is when you are from school very tired and at times you have to go and work a bit so you end up forgetting.” [P23, male]
	Needing airtime to call peer mentors	“Whenever I was in need of some inquiry I had to wait for the peer mentor to call.” [P30, male]
**Length of calls**		
	Calls were too short	“We talk for just 15 mins.... I would have liked to talk for 1 hour.” [P20, female] “I just wanted some extension in time, but she was always busy.” [P27, female]

###### Facilitators of the Intervention

The app’s user-friendly design and capability for offline use were the key facilitators that aligned with the high adherence rates from our quantitative findings. Participants unanimously found the Kuamsha app enjoyable, useful, and easy to navigate. Furthermore, participants appreciated the app’s flexibility, which allowed them to engage with it at their convenience and without relying on an internet connection.

Engagement was further enhanced by the app’s culturally relevant content and interactive gamification elements. Users were particularly drawn to the narrative storytelling format, which they found highly relatable and effective in improving mood and engagement. The characters, especially the coach and bird, were notable favorites, providing guidance and resonating with the users’ experiences.

The important role played by peer mentors was consistently recognized. Many adolescents viewed their peer mentors as not just guides but as friends who provided invaluable assistance and guidance. They also guided participants in using the app, providing tailored advice and reinforcing lessons from the app. This support was crucial in fostering engagement, with quantitative data indicating a surge in user interaction after peer mentor calls.

Finally, many participants appreciated the ability to plan the weekly calls in advance. They also highlighted that peer mentors were understanding and accommodating when changes were needed.

###### Barriers to the Intervention

Comprehension difficulties were noted as a key barrier, with some participants attributing this to literacy challenges and others mentioning that low mood at times affected their ability to understand the app’s content. This barrier might explain the varied engagement levels observed, suggesting that some adolescents might have benefitted from additional support, such as simplified language or audio aids.

Several participants experienced some technological issues during the intervention. For example, participants reported that stories would occasionally restart. We believe that the proximity of the “restart story” and “continue with story” options may have led to some confusion, causing participants to inadvertently restart stories when they intended to pick up where they left off. In addition, there were incidents where participants unintentionally deleted the app, requiring follow-up home visits.

Logistical issues with scheduling peer mentor calls were another barrier, with some participants finding it difficult to align calls with their personal schedules, including school and work. Although peer mentors were in charge of initiating the calls, some participants also mentioned that they would have liked to have the option to reach out to their mentors directly, a functionality that was not provided primarily due to practicality and resource constraints.

The feedback on call durations with peer mentors highlights varying needs among participants. While many found the 15-minute limit satisfactory, a few adolescents expressed a need for longer discussions. Some peer mentors also raised this as a barrier, particularly for adolescents requiring additional support. This variation aligns with the variability observed in engagement metrics, indicating that a uniform approach to peer support may not fully meet the diverse needs of all participants.

#### Fidelity of Delivery of the Intervention

##### Overview

Independent raters evaluated and rated 33.1% (49/148) of calls made by peer mentors. Of these, 63% (31/49) calls achieved the minimal adherence criteria of ≥90%, while a higher proportion, 94% (46/49), reached an adherence of ≥80%.

This indicates a generally high level of adherence among peer mentors though it also highlights a gradient in the adherence rates, with a substantial portion of calls not reaching the highest benchmark set for the study. Furthermore, an examination of the data reveals that 1 peer mentor’s average adherence was significantly lower than that of the other 3 mentors (*P*<.001), which adversely affected the overall average adherence.

In terms of competence, peer mentors achieved a relatively high average competency score of 75.8% (SD 5.7%) before starting with study participants.

##### Secondary Outcome Measures: Changes in Mental Health

Paired *t* tests did not reveal statistically significant differences in depressive symptoms (*P*=.16), emotional well-being (*P*=.90), and anxiety levels (*P*=.72) before and after the intervention (Table 7). However, the observed directional changes, particularly in the PHQ-A, indicate a promising trend in the expected positive direction, suggesting a potential benefit from the intervention, despite the lack of statistical significance. When examining the effect sizes, the PHQ-A also showed a small effect size (Cohen *d*=0.30). Supporting this trend, we also observed a reduction in the proportion of participants with moderate and moderately severe depressive symptoms (PHQ-A≥10) from 32% (10/31) to 17% (5/29) after the intervention, but this change was also not significant (*P*=.10).

**Table 7:** Pre- and postintervention results for the Kuamsha program (n=29).

	Baseline, mean (SD)	Endline, mean (SD)	Cohen *d* (95% CI)	Mean difference (95% CI)	*P* value
Depression symptoms: PHQ-A^a^ score	6.72 (4.77)	5.31 (4.77)	0.30 (–0.22 to 0.81)	1.41 (–0.60 to 3.42)	.16
Emotional well-being: WEMWBS^b^ score	48.28 (12.06)	47.93 (13.06)	0.03 (−0.49 to 0.54)	0.34 (−5.17 to 5.86)	.90
Anxiety symptoms: GAD-7^c^ score	4.55 (4.78)	4.21 (5.50)	0.07 (−0.45 to 0.58)	0.34 (−1.62 to 2.31)	.72

^a^PHQ-A: Patient Health Questionnaire Adolescent version.

^b^WEMWBS: Warwick-Edinburgh Mental Wellbeing Scale.

^c^GAD-7: Generalized Anxiety Disorder-7.

These findings point to the intervention’s potential for positively impacting mental health, with particular benefits for depression. Further research with a larger sample size is needed to assess the potential benefits of the intervention and how this differs depending on the level of engagement with the intervention.

## Discussion

### Principal Findings

This study presents findings on the feasibility and acceptability of the Kuamsha program, a culturally tailored digital intervention for Ugandan adolescents. The digital platform delivers behavioral activation therapy through an interactive narrative gaming smartphone app, complemented by low-intensity, phone-based guidance from trained peer mentors.

The results suggest that the Kuamsha program is a feasible and acceptable intervention to support adolescent mental health in Uganda. Notably, the study achieved excellent retention rates exceeding 90% and treatment adherence rates surpassing 80%, with almost half (14/31, 45%) of the participants continuing to use the app after the intervention. Our results, evaluated against our predefined trial progression criteria, indicate a successful feasibility study, with all criteria meeting the green region thresholds.

Qualitative data from the in-depth interviews and data from an acceptability questionnaire reinforce the quantitative findings on the intervention’s acceptability. Participants consistently described the app as engaging, useful, and user-friendly. The use of storytelling techniques and game design elements seemed to have enhanced the user experience, as was the fact that stories were relatable to our target population. In addition, the flexibility and offline functionality of the app made it highly accessible and easier for participants to incorporate the intervention into their daily lives.

Triangulation of quantitative and qualitative data also provided insights into barriers and areas for improvement. For example, while the app achieved high engagement metrics, interviews revealed barriers related to comprehension difficulties and technological glitches. While the peer mentors supported adolescents in solving some of these issues, these glitches highlighted the need for further user testing and robust technical support to ensure a smoother user experience. These barriers offer insights that complement the quantitative data, suggesting areas for improvement.

Participants found that peer mentors’ approachability, guidance, and communication skills played a pivotal role in enhancing the Kuamsha program experience. Qualitative findings also revealed that a subset of participants would have preferred a more extended interaction with peer mentors, a point that was also raised by peer mentors themselves. Such findings suggest that a more tailored intervention model where the intensity and duration of support are adaptable to individual needs might be more effective. This tailored model could range from the stand-alone app for those who require less support to extended peer mentorship for those who benefit from more in-depth interaction to even more personalized in-person sessions for those with the greatest needs. We plan to explore a more customized intervention approach in future studies.

Notably, the triangulation of data highlighted a difference between adolescents’ positive experiences with their peer mentors and the quantitative evaluation of intervention fidelity. Although adolescents reported very positive interactions, quantitative fidelity assessments revealed that only 63% (31/49) of peer mentor calls met the 90% adherence criterion. While most rated calls, 94% (46/49), reached an adherence of ≥80%, several interpretations may explain this discrepancy. First, this may suggest that peer mentors’ performance varied, with 1 mentor in particular not adhering to the protocol. Second, the ambitious 90% adherence threshold may have been overly optimistic, potentially overlooking the realities of implementing such interventions in a real-world setting. Third, the fidelity criteria may not have adequately captured the softer skills involved in building trust and rapport, which were highly valued by the adolescents. This indicates that the structure of the peer mentor program might have been too rigid, limiting mentors’ ability to provide emotional support.

The study’s secondary objective was to explore changes in mental health, specifically depressive and anxiety symptoms and emotional well-being. While we cannot draw quantitative conclusions due to the study’s design, qualitative findings suggest that some participants perceived improvements in mood and well-being.

### Comparison to Prior Work

Our study contributes to the limited research on digital psychological interventions designed specifically for school-age adolescents in sub-Saharan Africa, a population for whom few targeted interventions exist [48-50]. Our findings provide important insights into the scalability and acceptability of digital mental health solutions for Ugandan adolescents, a population largely overlooked in existing research.

Consistent with previous studies, our results highlight the importance of co-designing interventions with adolescents to enhance engagement and acceptability [22]. The narrative-gamified approach, which relies on storytelling and gaming elements, proved promising, with app engagement metrics—such as time spent on the app and modules completed—surpassing benchmarks from prior research [17,19,51-53].

Furthermore, supporting the app with low-intensity telephonic guidance significantly enhanced user engagement, aligning with evidence on the critical role of human support in digital interventions [18,54-56]. Our findings also suggest that relying on peer mentors is highly acceptable, offering a scalable model to treat common mental health disorders, particularly in settings with limited access to clinicians.

The observed trends in our mental health outcomes are consistent with the mixed results commonly reported in studies of digital interventions [18,57-59]. This variability may, in part, be attributed to the substantial heterogeneity in user engagement. Both our findings and prior research suggest that tailoring interventions to the specific needs of individuals could improve overall outcomes and enhance cost-effectiveness [60-62].

### Strengths and Limitations

This study has several strengths. First, it targets an often-overlooked population, adolescents in sub-Saharan Africa, who face significant mental health challenges with limited support [63,64]. Second, the Kuamsha app was developed through an iterative, user-centered design process with adolescents and key stakeholders to ensure cultural relevance and usability [25]. Third, the intervention’s integration of narrative and game design with evidence-based behavioral activation therapy, supported by phone-based peer mentor guidance, created an engaging and scalable therapeutic model. Finally, feasibility and acceptability were rigorously assessed using mixed methods and comprehensive app engagement metrics, all evaluated against predefined trial progression criteria [42].

Several limitations are noted. First, the sample size of the study was small, and the lack of statistically significant findings could be attributed to this. Changes in participants’ mental health were secondary outcomes, and the study was not powered to detect definitive treatment effects; thus, these results should be interpreted with caution. Second, this was a single-arm study; therefore, it is impossible to determine the direct impact of the intervention without a control group. The sister study in South Africa, which included an active control group, will provide further insights into the intervention’s efficacy as well as its feasibility among adolescents with depression [65]. Third, the formative work revealed literacy difficulties among some adolescents; therefore, a reading comprehension test was included as part of the screening criteria, which might have excluded the most vulnerable adolescents. To mitigate this, we attempted to limit the amount of text in the app, and all language was co-designed and thoroughly tested with adolescents to ensure it was accessible. Further app development work should explore the use of audio voiceovers or alternate features to increase the accessibility of the app to low-literacy populations. Fourth, we encountered logistical challenges in rolling out the intervention, including inconsistent mobile coverage and limited access to charging stations, as not all adolescents had a reliable place to charge their phones. To address these, we designed the app to operate primarily offline and distributed solar chargers where needed. While these measures helped reduce study disruptions, they may pose challenges for scaling. In future iterations, we will explore options for optimizing the app’s battery efficiency and establishing partnerships with local mobile providers to ensure the intervention is accessible and effective for all users. Finally, the COVID-19 pandemic posed significant challenges to the execution of this study, impacting both recruitment processes and the manner in which the intervention was delivered. Study participants were enrolled during a period of global uncertainty, which sometimes led to delays or modifications in our procedures (eg, dropping the SMS text message–based symptom monitoring). The study design did not allow us to isolate the pandemic’s effects from the intervention’s effects.

### Future Directions

There is an urgent need for scalable psychological treatments to support adolescent mental health in low-income countries like Uganda. The findings from this study also suggest that the Kuamsha program may be adaptable to other low-income settings with similar sociocultural and socioeconomic contexts. Key features, such as offline functionality and low-intensity peer mentor integration, support its scalability across diverse sub-Saharan African regions, including rural South Africa, where a sister study was also conducted.

Digital mental health interventions are promising, but few have been developed explicitly for use or rigorously evaluated with adolescents in sub-Saharan Africa. The Kuamsha program, demonstrated here as a feasible and acceptable intervention for Ugandan adolescents, has the potential to fill this gap. The insights gathered through this study will inform the adaptation and enhancement of the intervention to ensure its effectiveness and accessibility in a fully powered randomization control trial. By addressing the identified limitations, the Kuamsha program could potentially offer a valuable addition to the landscape of adolescent mental health interventions in low-income settings.

## Appendix

Multimedia Appendix 1 Samples of interview guides.

Multimedia Appendix 2 Kuamsha app wireframes.

Multimedia Appendix 3 Distribution of responses to the Patient Health Questionnaire and the Generalized Anxiety Disorder tool at baseline.

Multimedia Appendix 4 Distribution of key app metrics.

## Abbreviations

GAD-7: Generalized Anxiety Disorder-7
PHQ-A: Patient Health Questionnaire Adolescent version
WEMWBS: Warwick-Edinburgh Mental Wellbeing Scale

## Data Availability

Deidentified data and replication files will be made available on the first authors’ personal website [66] after the publication of the study. The authors plan to make the Kuamsha app widely available, conditional on demonstrating effectiveness in a larger, fully powered trial. If effective, the app will be available for download on the Google Play Store once that trial is complete.
