# Supplementary material for: A Narrative-Gamified Mental Health App (Kuamsha) for Adolescents in Uganda: Mixed Methods Feasibility and Acceptability Study
Source: JMIR Serious Games. 2024 Dec 19;12:e59381. doi: 10.2196/59381 (PMC11695961; doi:10.2196/59381)
Supplement: Multimedia Appendix 1 [file games_v12i1e59381_app1.docx]

# Supplementary materials – A narrative-gamified app to support adolescent’s mental health in Uganda: a mixed-methods feasibility and acceptability study

## Multimedia Appendix 1

Samples of interview guides

1. **What did you think about using the app?**
   1. Did you find it enjoyable/ unenjoyable? Why?
2. **What did you like most about the app? What did you like least about the app?**
   1. What story did you choose? Why?
   2. Were there any characters that reminded you of yourself/ other people in your life?
   3. What did you think about the bird character that gives advice?
   4. What was your favorite character? Why?
   5. Do you still remember the activities you had to do each week? What did you think about these activities? Did you ever change the activities after speaking to your Peer Mentor?
   6. Where there any lessons from the app that you could apply to your own life?
   7. What, if anything, would you change about the app?
3. **How did you use the app?**
   1. Did only you use the app or did you share it with others?
   2. Did other people (family/ friends) know you were using the app? How did that make you feel?
   3. How often did you use the app/ how much time did you spend on it?
   4. What things made it easier/ more challenging to use the app?
4. **What did you think about having a Peer Mentor?**
   1. Was it easy/ challenging to find a time to talk to the Peer Mentor each week? Why/ why not?
   2. Tell me a bit about your relationship with your peer mentor? Did you feel comfortable talking with him/ her? Why/ why not?
   3. Did the Peer Mentor calls change the way you used the app? If so, how?
   4. What do you think of the combination of using the app and receiving phone calls? Would you have preferred to only have one of those (i.e. app only or phone calls only). Why / why not?
   5. What, if anything, would you change about the Peer Mentor calls?
5. **What did you think about getting a phone?**
   1. How did you use the phone – did only you use it, or did you share it with other people?
   2. Did getting a phone change the way other people (parents/ family/ friends) saw you? How?
6. **What expectations did you have about participating in this study**
   1. Did the study meet/ not meet your expectations? Why/ why not?
   2. What would have improved your experience of participating in this study?
   3. Would you recommend participating in the study to other young people? Why/ why not?
   4. Is there anything else you would like to share about your experience in this study?
