# Supplementary material for: A Narrative-Gamified Mental Health App (Kuamsha) for Adolescents in Uganda: Mixed Methods Feasibility and Acceptability Study
Source: JMIR Serious Games. 2024 Dec 19;12:e59381. doi: 10.2196/59381 (PMC11695961; doi:10.2196/59381)
Supplement: Multimedia Appendix 2 [file games_v12i1e59381_app2.docx]

# Supplementary materials – A narrative-gamified app to support adolescent’s mental health in Uganda: a mixed-methods feasibility and acceptability study

## Multimedia Appendix 1

Table A1. Kuamsha app wireframes

| App component | Description | Screenshoot |
| --- | --- | --- |
| Log-in unlock code | Kuamsha is password-protected. Users need to enter a password every time they access the app. | 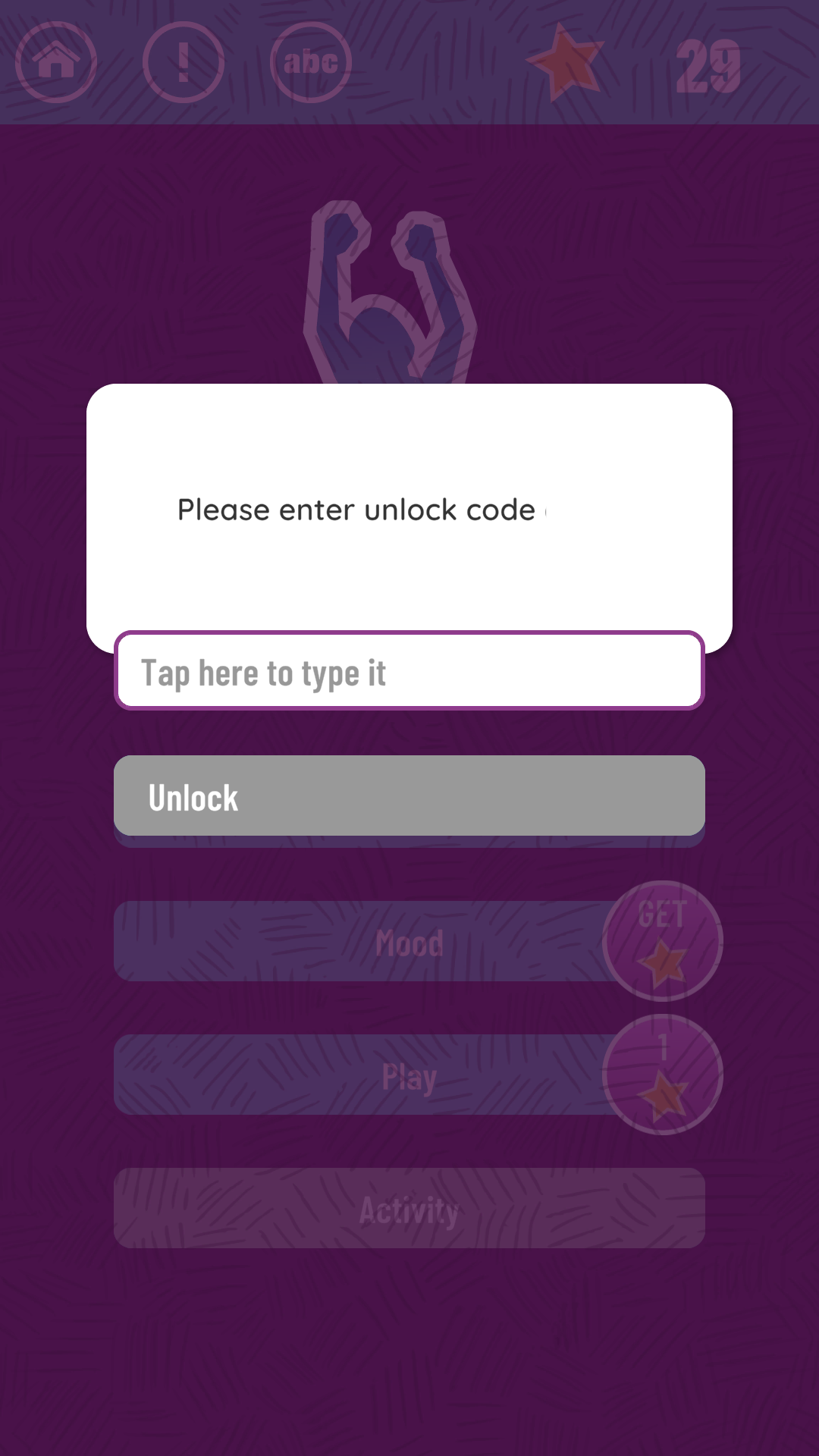 |
| Home screen | This is the first screen that users see as they log-in to the Kuamsha app. Users have the option to play through the stories, monitor their mood, play absorbing activities to improve focus, or report on their weekly activities (see below for further details on each of these components). | 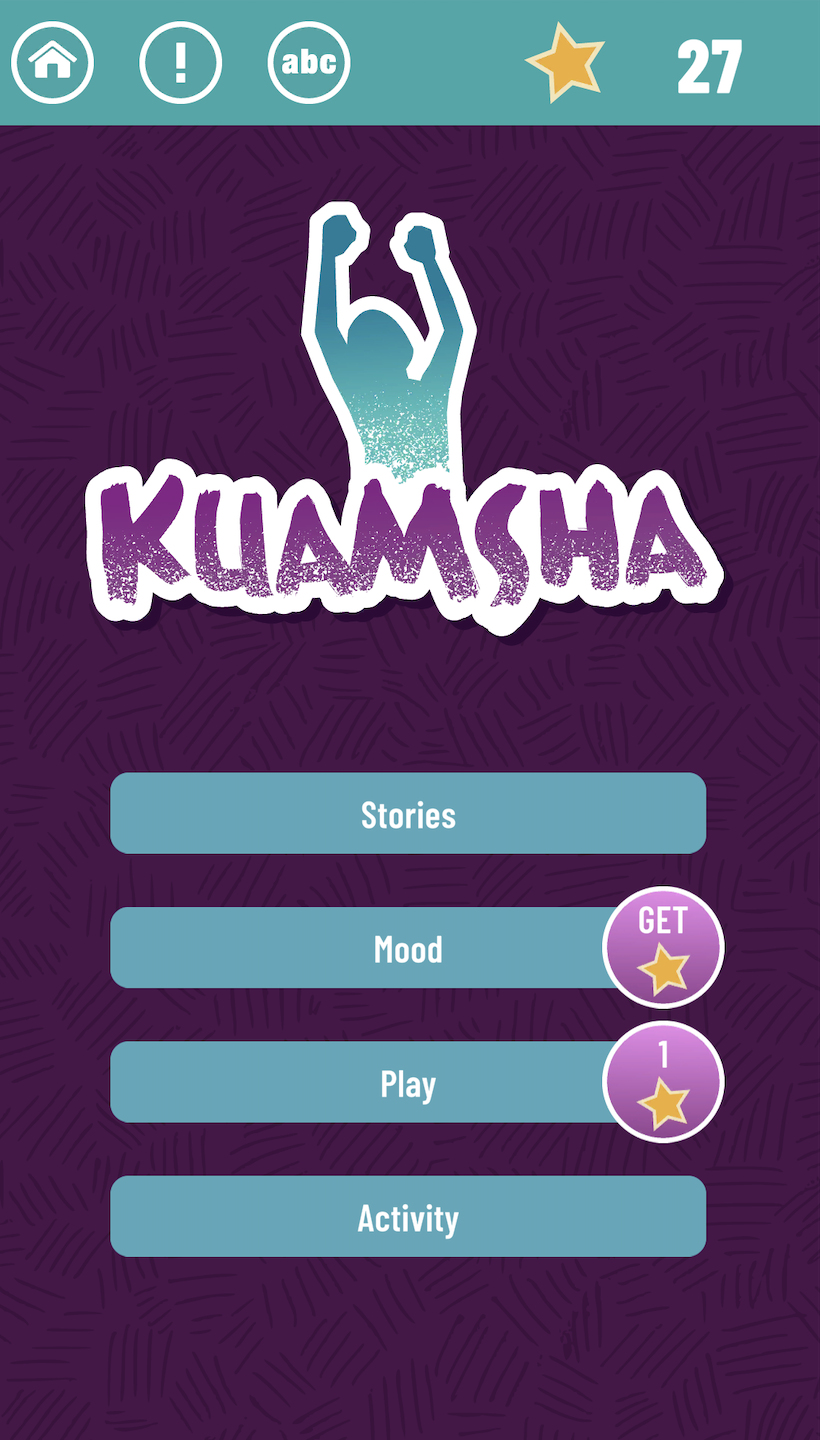 |
| Onboarding process | When users open the app for the first time they are introduced and guided through its main components. This onboarding process aims to teach adolescents how to interact with the interface, choose their preferred language, locate the emergency button, and select one of the stories. | 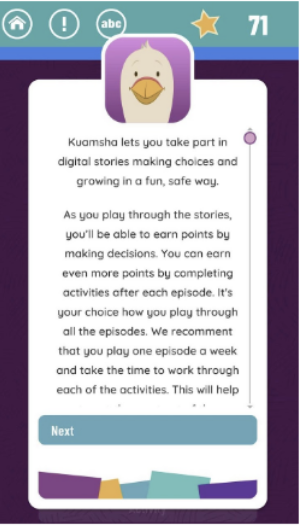 |
| Language selector | Users can select their preferred language. All of the text in the app underwent two rounds of translation and has been checked by a clinical psychologist for accuracy. | 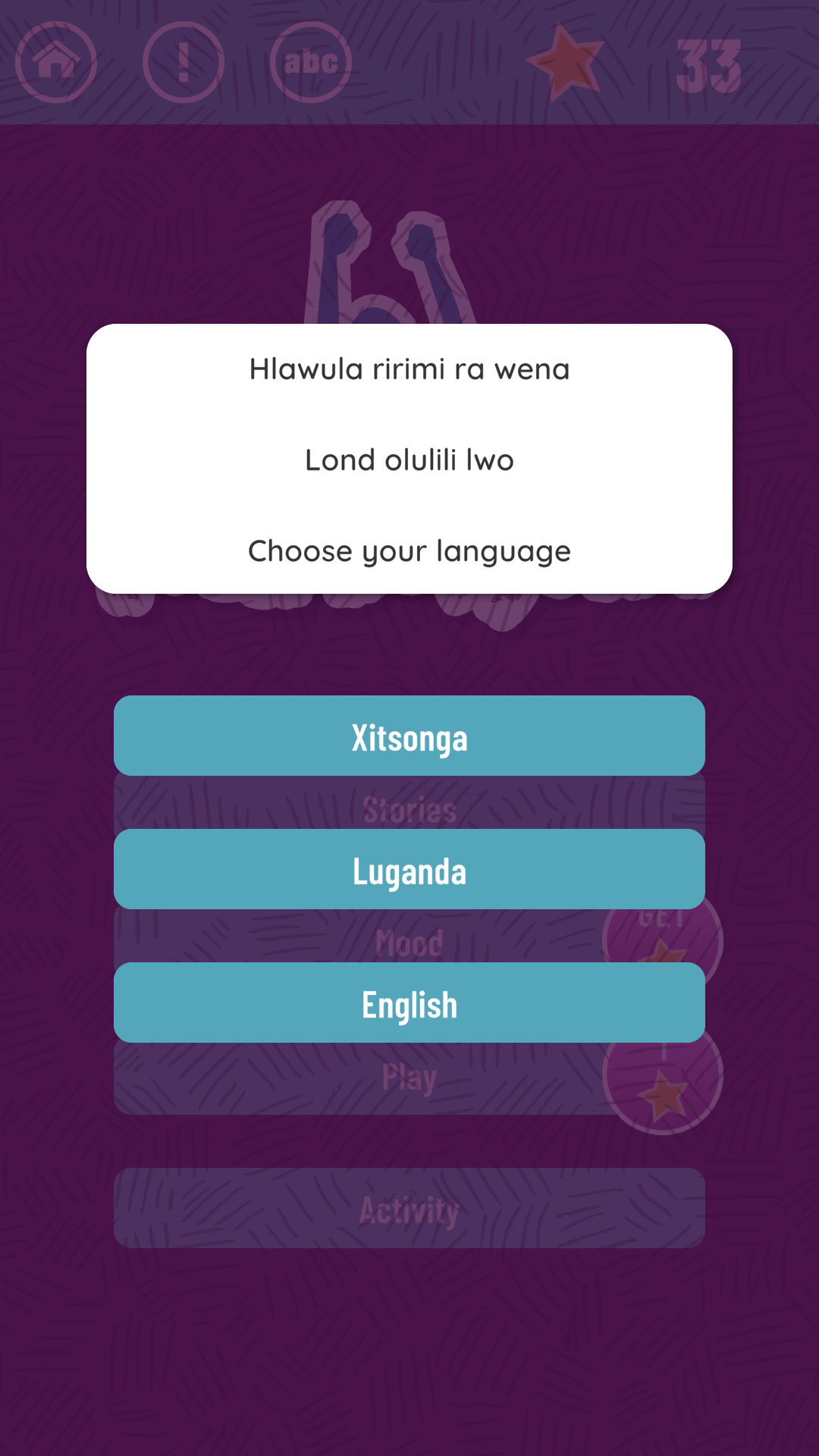 |
| Story Selection | The core of the game consists of a choice between two narrative stories. Each consists of six modules that are played in sequential order. It is possible to begin one story and then switch to the other. During gameplay points are earned for the choices made and by completing other core game elements described below. | 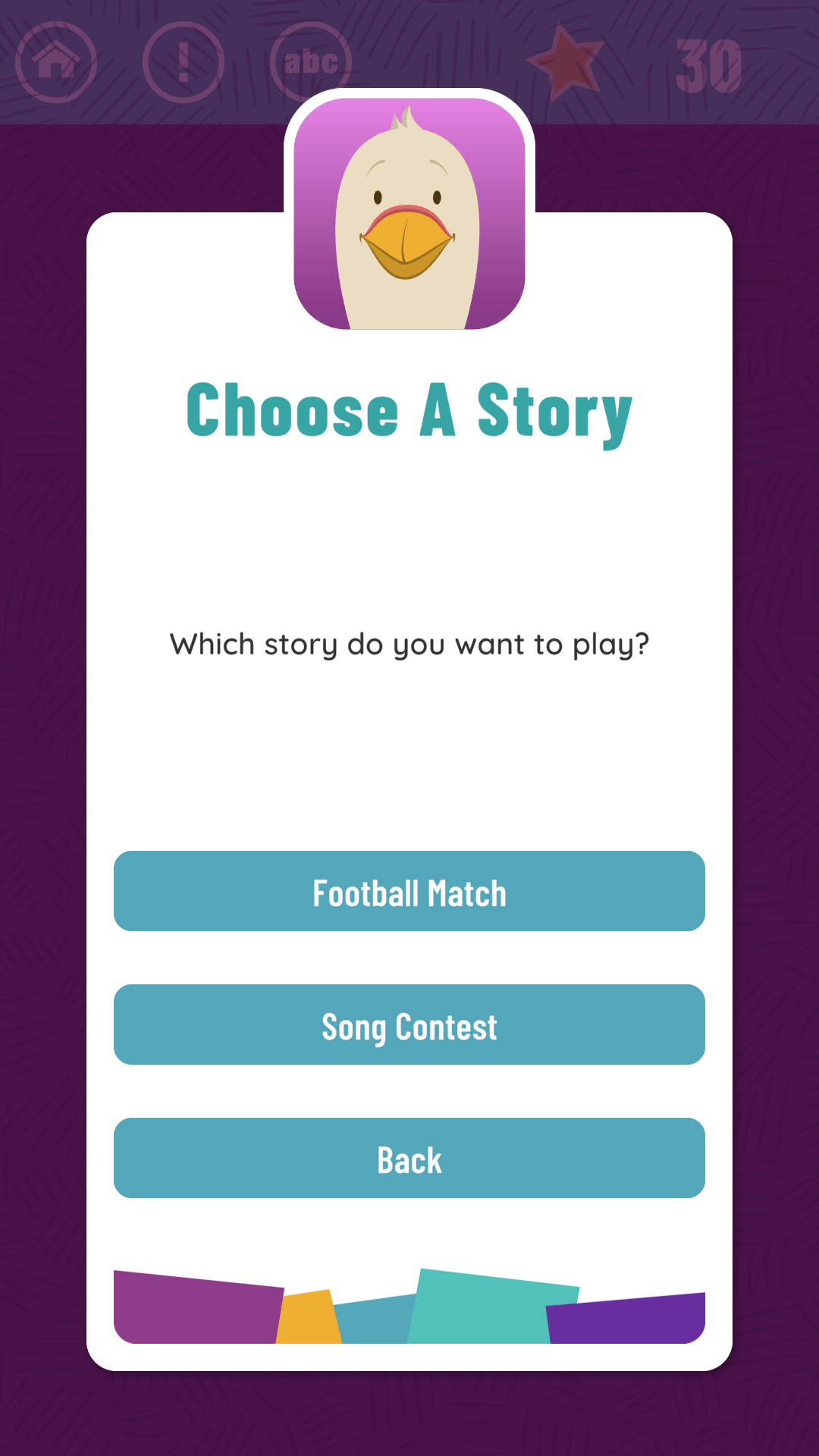 |
| Choices affect outcomes | A key principle of the game is that the player is drawn into the narrative through the use of interactive choice. Choices made by the player branch the story in ways that lead the character down different pathways in the game. | 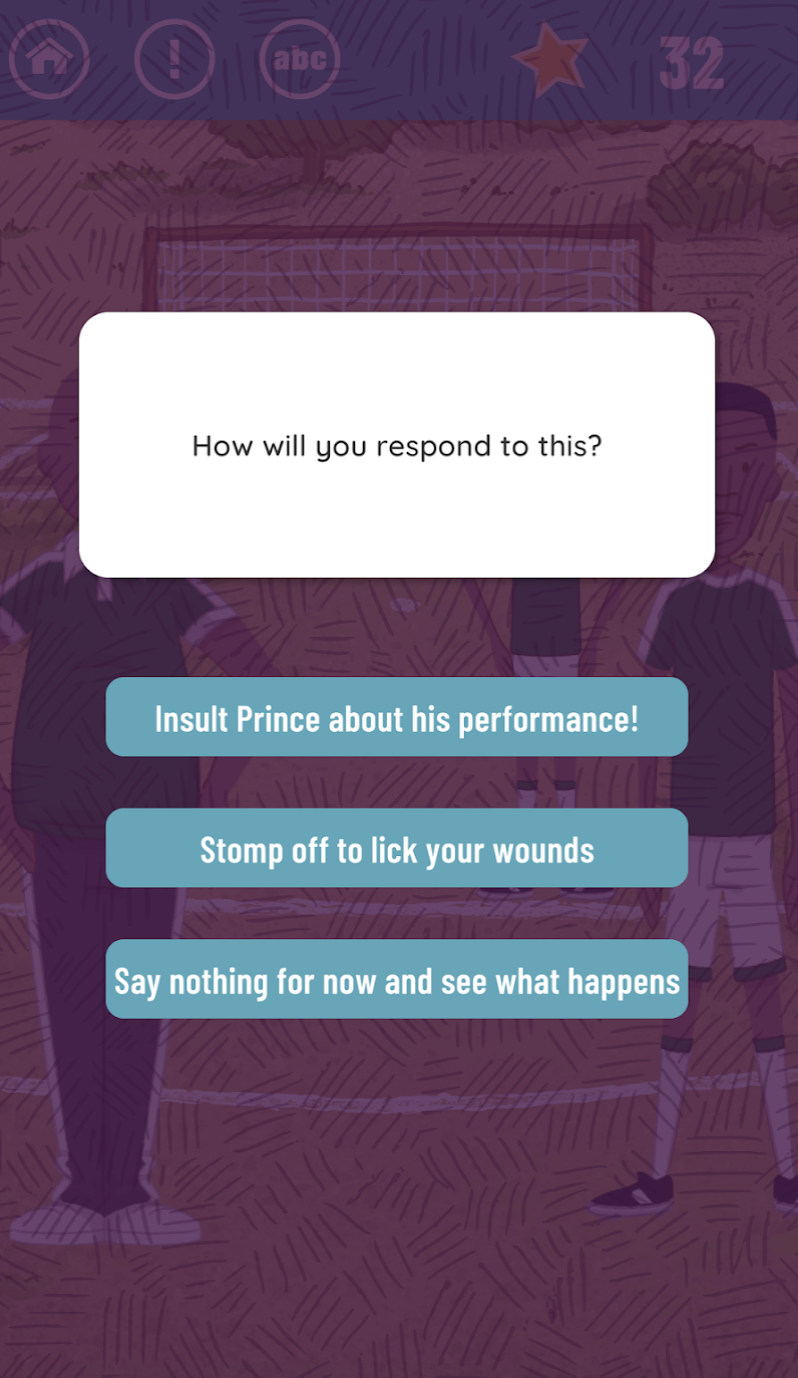 |
| Bird Guide | The bird character is used throughout the game as a way of having players reflect on their choices, supporting review of what as been learnt, offering suggestions and other game mechanics designed to draw the multiple components of the game into a unified whole. | 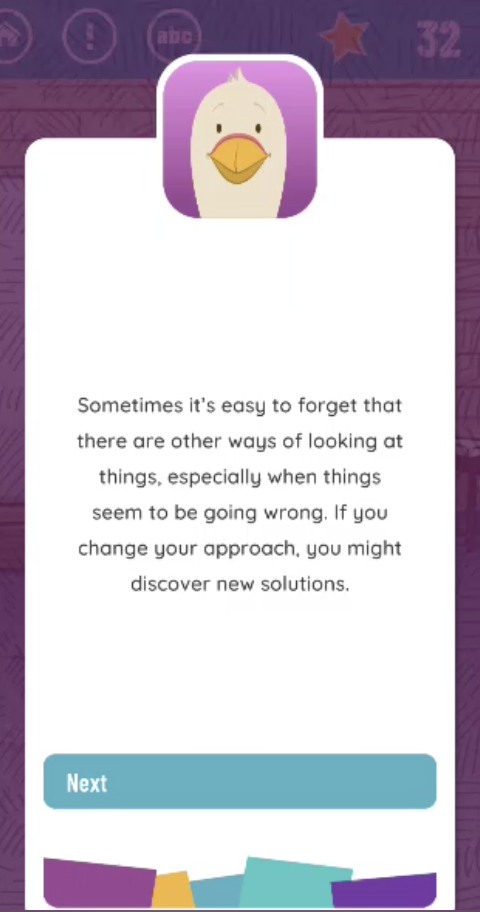 |
| Summary of lessons learned | In order to ensure that players are reflecting on the game and show some level of understanding of the concepts and choices made, each story episode ends with a summary of lessons learned. | 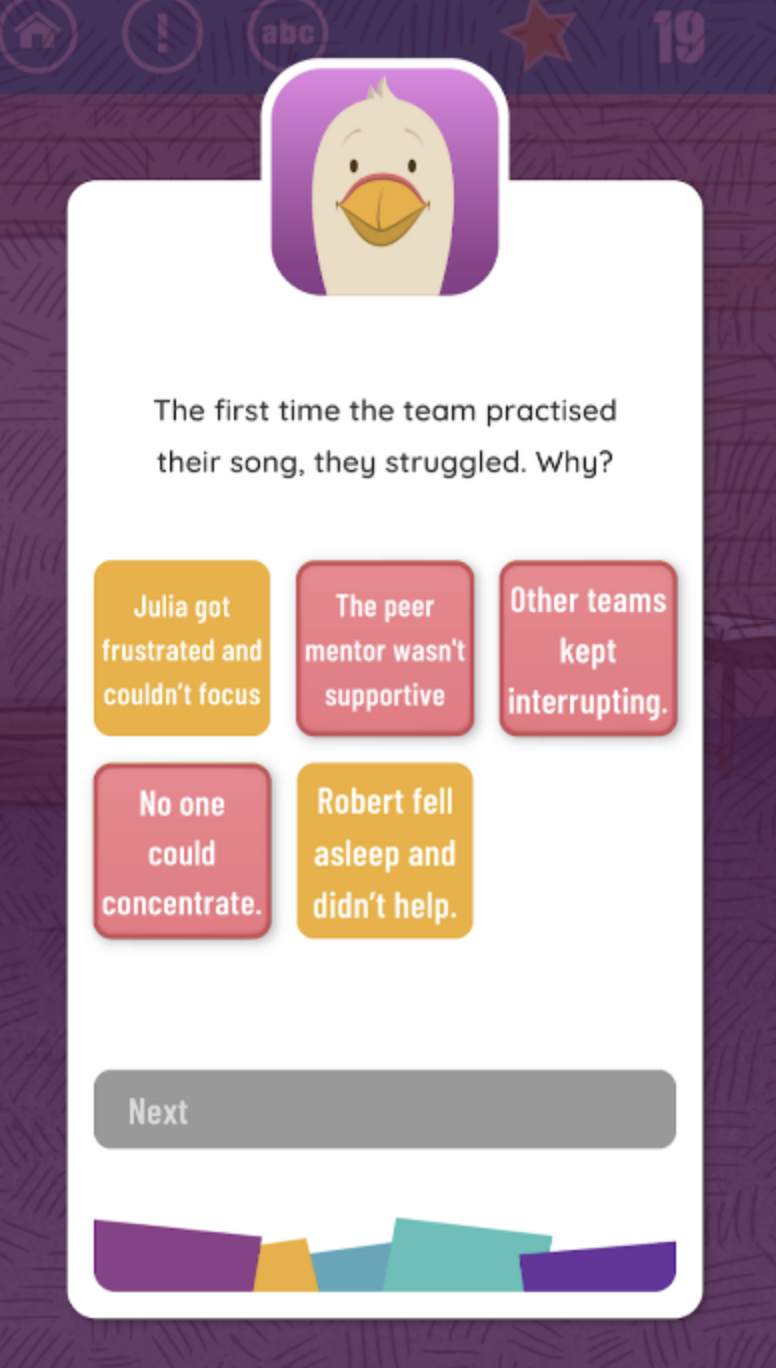 |
| Real-life exercises | Users are asked to think about an activity that they could do that is associated with the principle of the episode they just completed. Each homework session consists of one or two activities that expand on the Behavioral Activation principle covered within that session. | 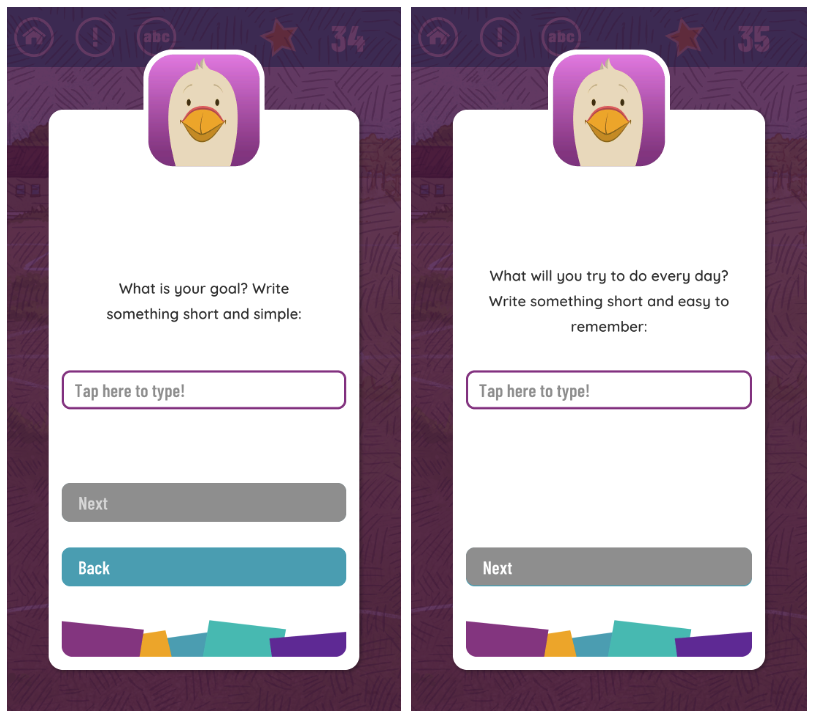 |
| Mood monitoring | Participants are asked to monitor their mood different times (before episode and after episode, when they report homework, when they complete absorbing activity). Users get feedback on how their mood changes over time and in-app points every time they complete it. | 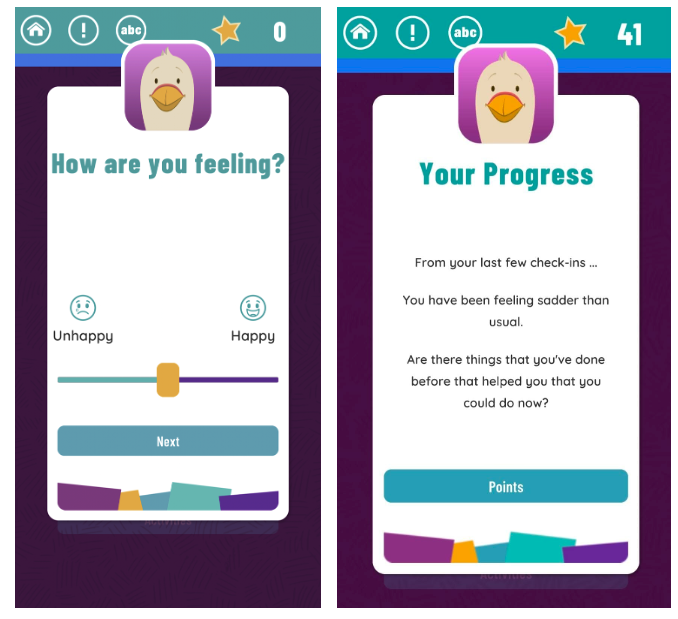 |
| Notifications | Users are reminded to report their progress on their weekly activities via notifications. | 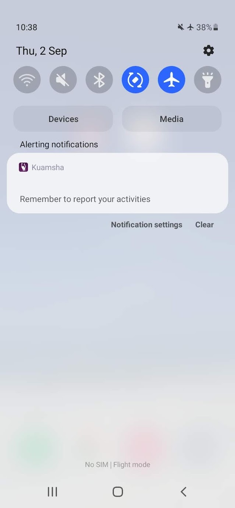 |
| Game design elements:  personalization | Users are asked to personalize their character in the story by choosing their name, preferred pronouns, and their team’s name. | 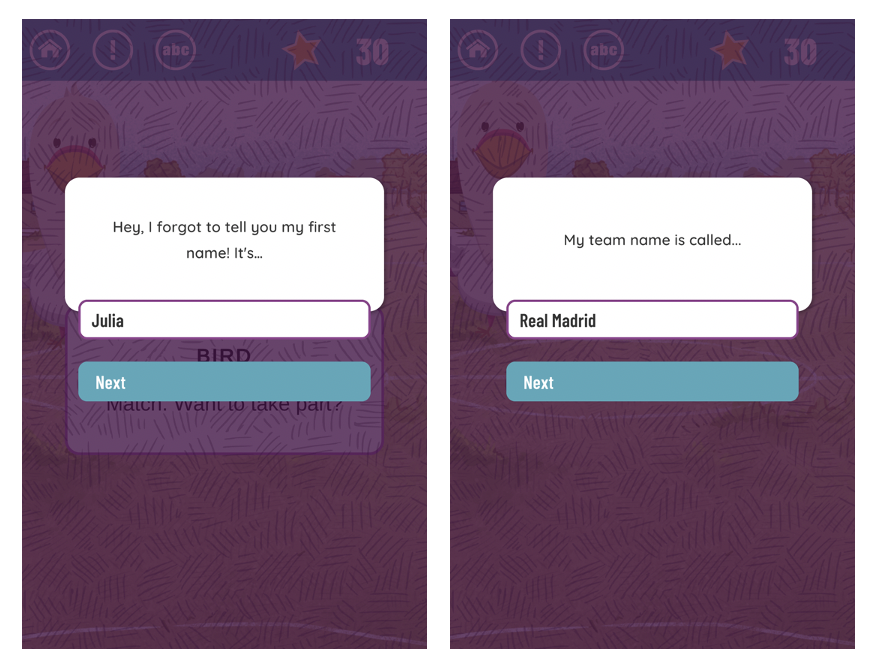 |
| Game design elements:  In-app points | Participants earn in-app points every time they complete an episode, report their weekly activity, monitor their mood, and play the absorbing activities. | 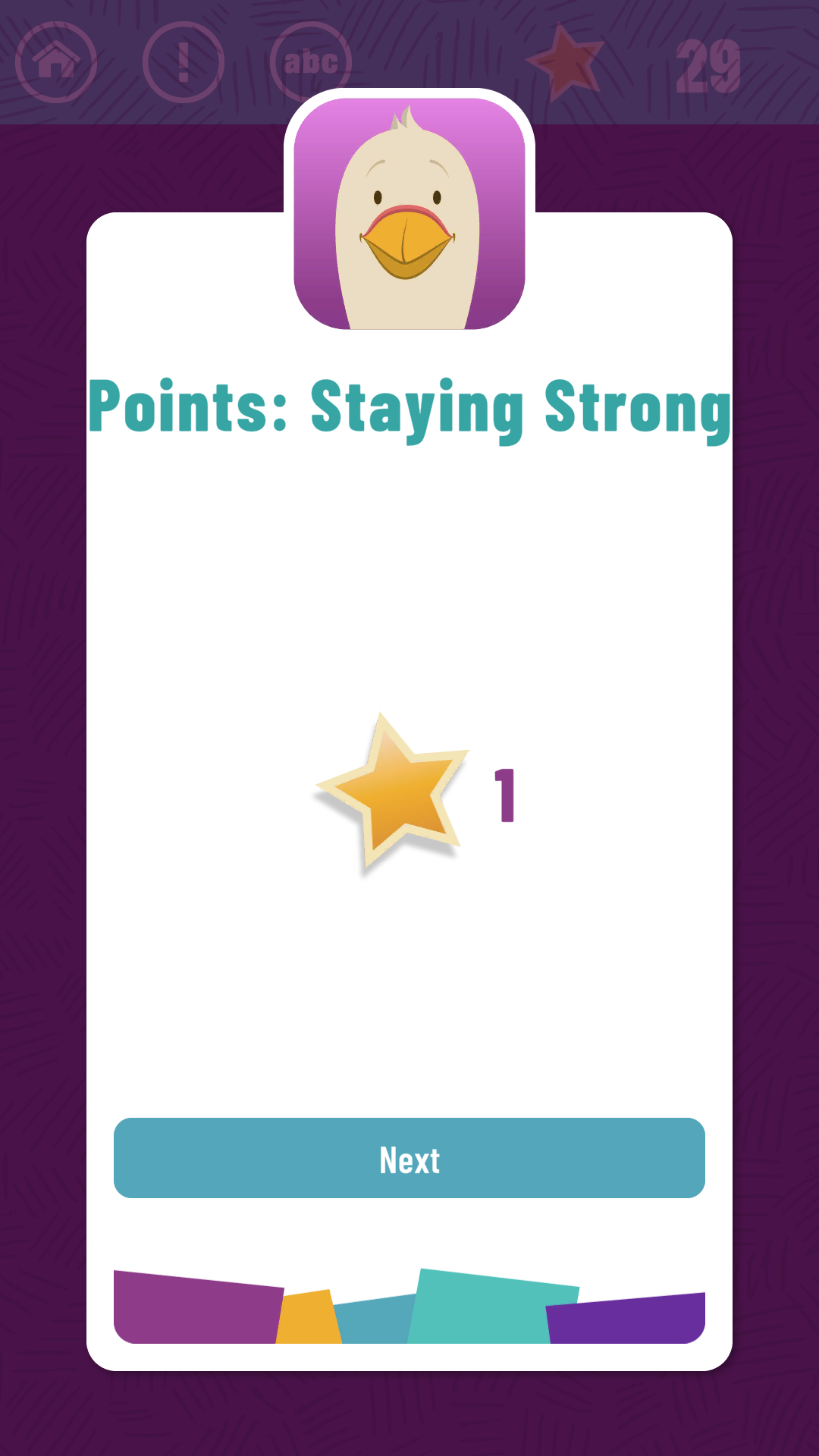 |
| Game design elements:  Absorbing activities | Kuamsha includes two different activities to teach the concept of absorption (focusing on an activity to detract from negative thoughts). Participants can decide between two different absorbing activities: a music-absorbing activity (rhythm game where users tap the screen in time with the music) and a football-absorbing activity (users practice at taking shots on goal). | 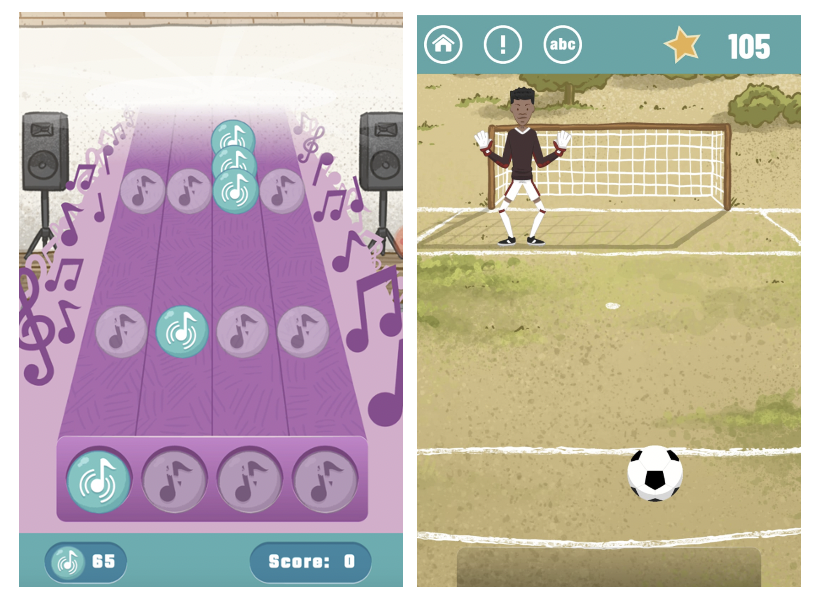 |
| Emergency button | Kuamsha includes an “emergency button” for adolescents to call in event that they are feeling extremely sad or thinking about hurting themselves. | 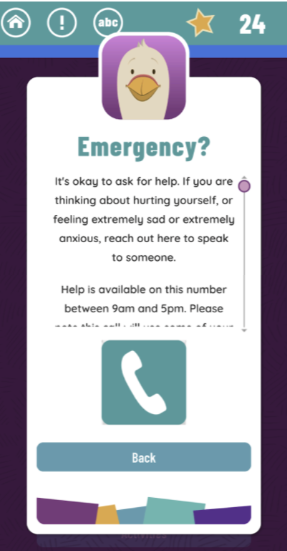 |
| Examples of app visuals | | |
| \|  \| \|  \| \| \| --- \| --- \| --- \| --- \| \|  \| \|  \| \| \| 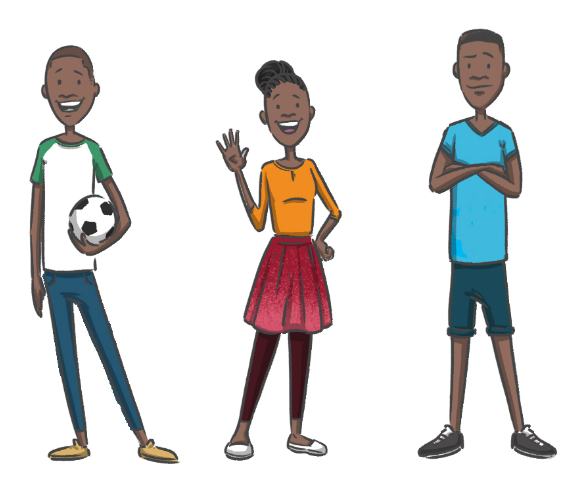 \| 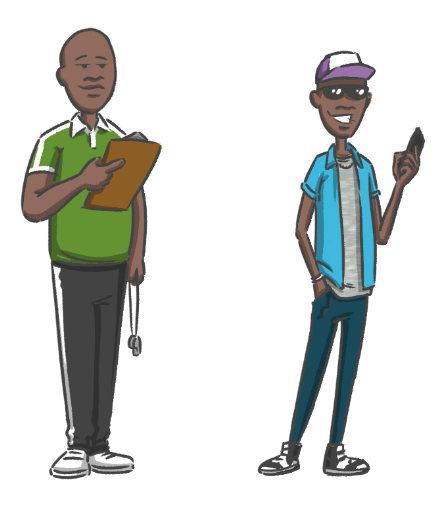 \| \| 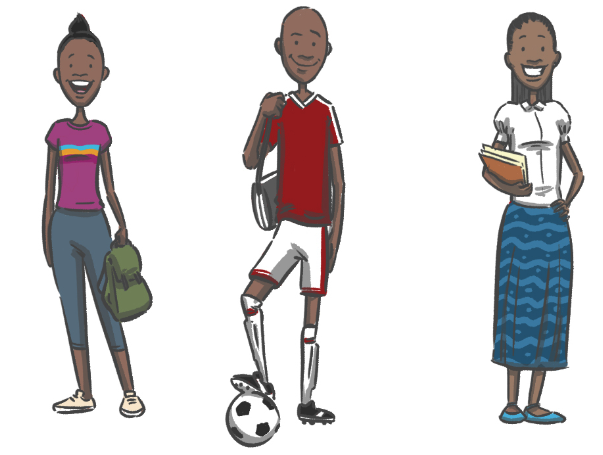 \| \| Story characters \| \| \| \| | | |
| \| 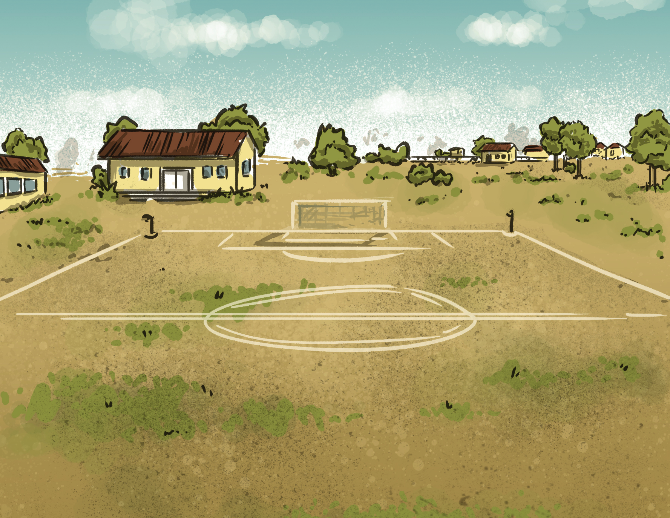  Football Pitch \| 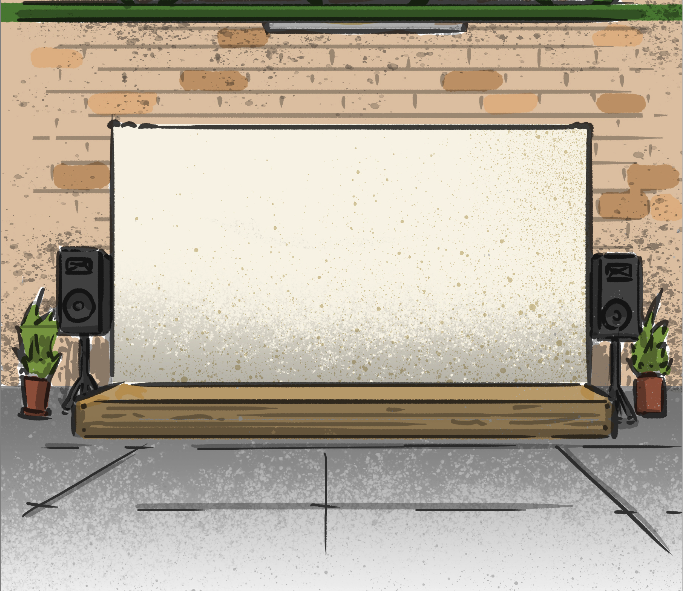  Song Contest Stage \| \| --- \| --- \| \| 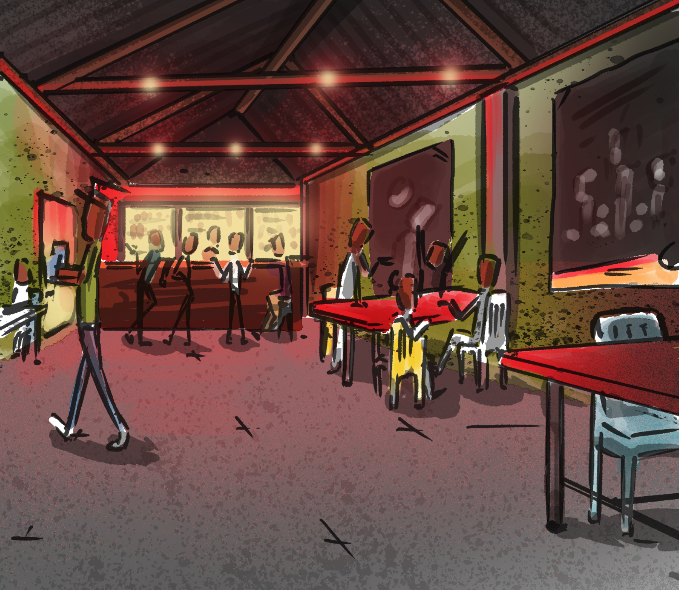  Tavern \| 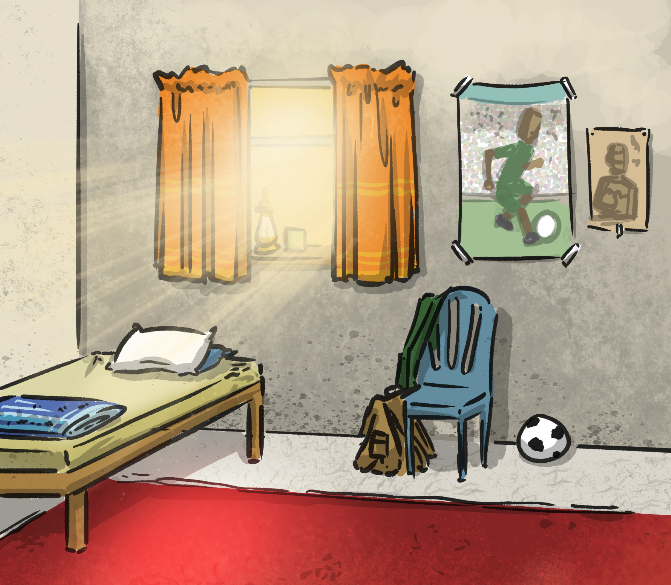  Main character’s room \| | | |
