# Supplementary material for: A Narrative-Gamified Mental Health App (Kuamsha) for Adolescents in Uganda: Mixed Methods Feasibility and Acceptability Study
Source: JMIR Serious Games. 2024 Dec 19;12:e59381. doi: 10.2196/59381 (PMC11695961; doi:10.2196/59381)
Supplement: Multimedia Appendix 3 [file games_v12i1e59381_app3.docx]

# Supplementary materials – A narrative-gamified app to support adolescent’s mental health in Uganda: a mixed-methods feasibility and acceptability study

## Multimedia Appendix 2


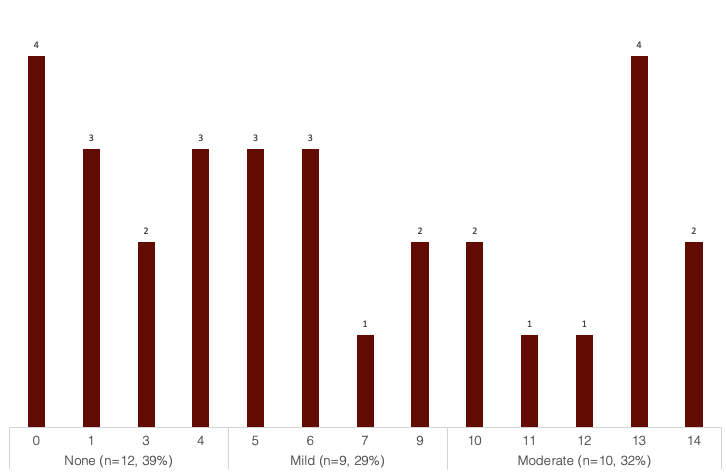


Distribution of the Patient Health Questionnaire (PHQ) at baseline


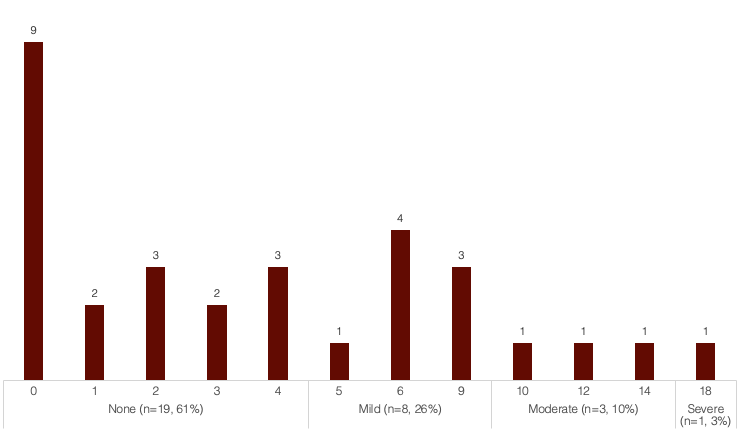


Distribution of the Generalized Anxiety Disorder (GAD-7) at baseline
