# Supplementary figures and images for: A Narrative-Gamified Mental Health App (Kuamsha) for Adolescents in Uganda: Mixed Methods Feasibility and Acceptability Study
Source: JMIR Serious Games. 2024 Dec 19;12:e59381. doi: 10.2196/59381 (PMC11695961; doi:10.2196/59381)

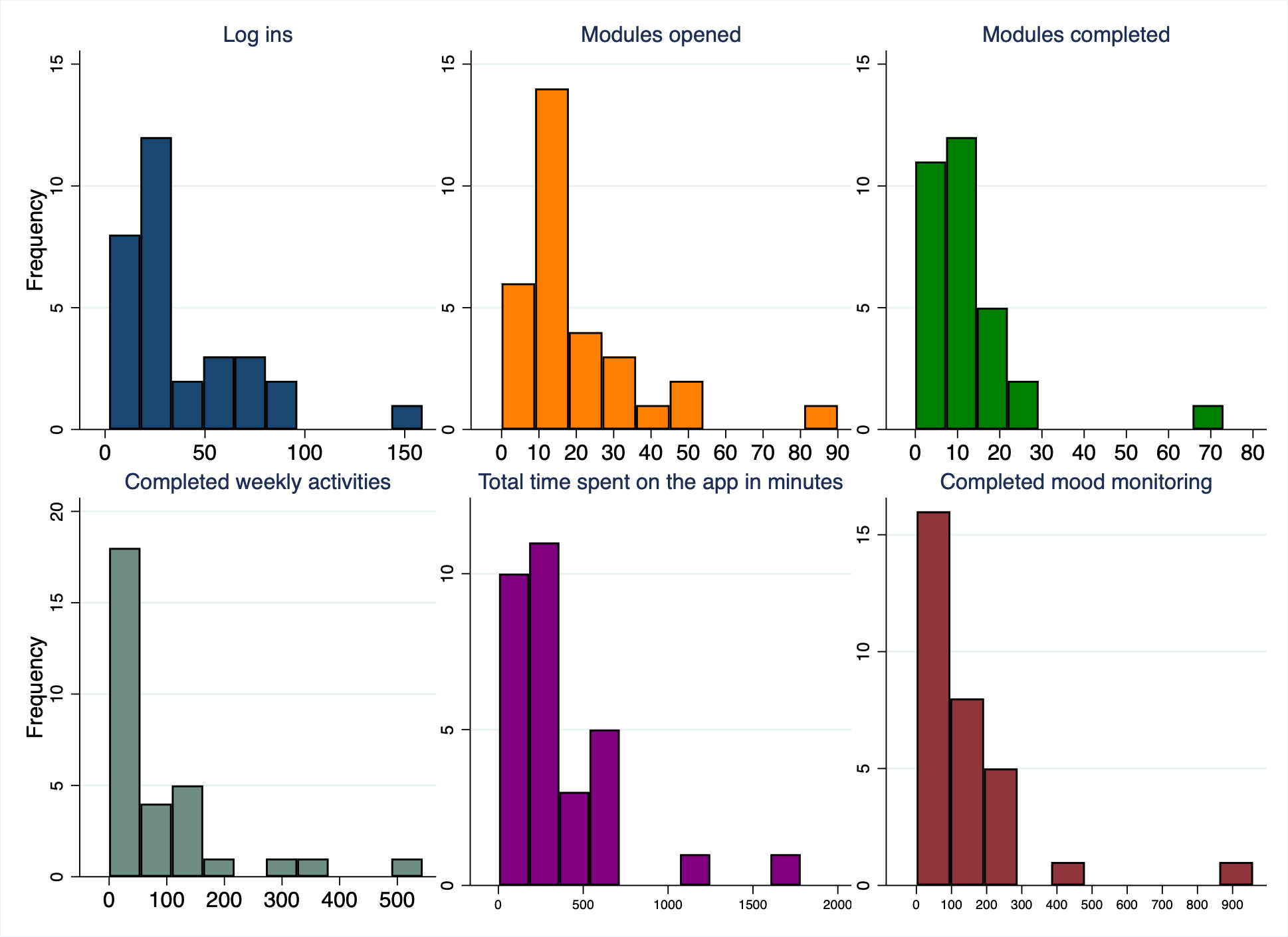

Supplement: Multimedia Appendix 4 [file games_v12i1e59381_app4.png]
